# Supplementary material for: Older adults’ attitudes towards deprescribing and medication changes: a longitudinal sub-study of a cluster randomised controlled trial
Source: BMJ Open. 2024 Jan 10;14(1):e075325. doi: 10.1136/bmjopen-2023-075325 (PMC10806786; doi:10.1136/bmjopen-2023-075325)
Supplement: Author's manuscript [file bmjopen-2023-075325.draft_revisions.pdf]

BMJ Open is committed to open peer review. As part of this commitment we make the peer review history of every article we publish publicly available.

When an article is published we post the peer reviewers' comments and the authors' responses online. We also post the versions of the paper that were used during peer review. These are the versions that the peer review comments apply to.

The versions of the paper that follow are the versions that were submitted during the peer review process. They are not the versions of record or the final published versions. They should not be cited or distributed as the published version of this manuscript.

BMJ Open is an open access journal and the full, final, typeset and author-corrected version of record of the manuscript is available on our site with no access controls, subscription charges or pay-per-view fees (http://bmjopen.bmj.com).

If you have any questions on BMJ Open's open peer review process please email info.bmjopen@bmj.com

BMJ Open

Association between patients' willingness to have medications deprescribed and changes in medication-related outcomes after one year

Journal:	*BMJ Open*
Manuscript ID	bmjopen-2023-075325
Article Type:	Original research
Date Submitted by the Author:	04-May-2023
Complete List of Authors:	Weir, Kristie; University of Bern; The University of Sydney<br>Jungo, Katharina Tabea; University of Bern<br>Cateau, Damien; University of Geneva, Institute of Pharmaceutical Sciences of Western Switzerland; University of Lausanne, Community Pharmacy, Center for Primary Care and Public Health (Unisanté)<br>Streit, Sven; University of Bern
Keywords:	Aged, Primary Health Care, Primary Care < Primary Health Care

SCHOLARONE™
Manuscripts

I, the Submitting Author has the right to grant and does grant on behalf of all authors of the Work (as defined in the below author licence), an exclusive licence and/or a non-exclusive licence for contributions from authors who are: i) UK Crown employees; ii) where BMJ has agreed a CC-BY licence shall apply, and/or iii) in accordance with the terms applicable for US Federal Government officers or employees acting as part of their official duties; on a worldwide, perpetual, irrevocable, royalty-free basis to BMJ Publishing Group Ltd ("BMJ") its licensees and where the relevant Journal is co-owned by BMJ to the co-owners of the Journal, to publish the Work in this journal and any other BMJ products and to exploit all rights, as set out in our licence.

The Submitting Author accepts and understands that any supply made under these terms is made by BMJ to the Submitting Author unless you are acting as an employee on behalf of your employer or a postgraduate student of an affiliated institution which is paying any applicable article publishing charge ("APC") for Open Access articles. Where the Submitting Author wishes to make the Work available on an Open Access basis (and intends to pay the relevant APC), the terms of reuse of such Open Access shall be governed by a Creative Commons licence – details of these licences and which Creative Commons licence will apply to this Work are set out in our licence referred to above.

Other than as permitted in any relevant BMJ Author's Self Archiving Policies, I confirm this Work has not been accepted for publication elsewhere, is not being considered for publication elsewhere and does not duplicate material already published. I confirm all authors consent to publication of this Work and authorise the granting of this licence.

For peer review only

Association between patients' willingness to have medications deprescribed and changes in medication-related outcomes after one year

Author list: Kristie Rebecca Weir* [1,2], Katharina Tabea Jungo* [1], PhD, PhD, Damien Cateau [3,4], PhD, Sven Streit [1], PhD
*shared first

Affiliations
[1] Institute of Primary Health Care BIHAM, University of Bern, Bern, Switzerland
[2] Sydney School of Public Health, Faculty of Medicine and Health, The University of Sydney, Sydney, Australia
[3] Community Pharmacy, Centre for Primary Care and Public Health (Unisanté), University of Lausanne, Lausanne, Switzerland
[4] School of Pharmaceutical Sciences, University of Geneva, Geneva, Switzerland

Corresponding author:
Kristie Rebecca Weir
Kristie.weir@unibe.ch
Mittelstrasse 43, 3007 Bern, Institute of Primary Health Care BIHAM, University of Bern, Bern, Switzerland

Word count manuscript: 2’774

Key words (5): polypharmacy, deprescribing, older adults, comorbidity, medications

For peer review only

For peer review only - http://bmjopen.bmj.com/site/about/guidelines.xhtml

Abstract

Objective: To investigate the association between older patients' willingness to have one or more medications deprescribed and: (1) change in medications, (2) change in the appropriateness of medications, and (3) implementation of prescribing recommendations generated by the electronic decision support system tested in the 'Optimizing PharmacoTherapy In the Multimorbid Elderly in Primary CAre' (OPTICA) trial.

Design: A longitudinal sub-study of the OPTICA trial, a cluster randomized controlled trial.

Setting: Swiss primary care settings.

Participants: Participants were aged ≥65 years, with ≥3 chronic conditions, and ≥5 regular medications recruited from 43 GP practices.

Exposures: At baseline, patients' willingness to have medications deprescribed was assessed using 3 questions from the 'revised Patient Attitudes Towards Deprescribing' (rPATD) questionnaire and its concerns about stopping score.

Measures/Analyses: Medication-related outcomes were collected at 1-year follow-up. Aim 1 outcome: change in the number of long-term medications between baseline and 12-month follow-up. Aim 2 outcome: change in medication appropriateness (Medication Appropriateness Index). Aim 3 outcome: binary variable on whether any prescribing recommendation generated during the OPTICA medication review was implemented. We used multilevel linear regression analyses (Aim 1, Aim 2) and multilevel logistic regression analyses (Aim 3). Models were adjusted for sociodemographic variables and the clustering effect at GP level.

Results: 298 patients completed the rPATD at baseline, 45% were women and 78 was the median age . A statistically significant association was found between the concerns about stopping score and the change in the number of medications over time (per 1-unit increase in the score the average number of medications use was 0.65 higher; 95% CI: 0.08 to 1.22). There was no statistically significant association between patients' willingness to have medications deprescribed and medication-related outcomes.

Conclusions: These findings indicate that there is no association between patients' willingness to have medications deprescribed and medication-related outcomes over one year.

For peer review only - http://bmjopen.bmj.com/site/about/guidelines.xhtml

Strengths and limitations

- This study investigated the association between older patients' willingness to have medications deprescribed and medication-related outcomes over time.
- This was a longitudinal sub-study of the OPTICA trial, which is a cluster randomized controlled trial conducted in Swiss primary care settings.
- Older adults agreeing to participate in the OPTICA trial could have had a higher willingness to have one or more of their medications deprescribed.
- Except for the statistically significant association between the concerns about stopping score and the number of medications over time, we did not find evidence for a statistically significant association between patients' willingness to have medications deprescribed and medication-related outcomes over time.
- A self-report questionnaire used to measure patients' hypothetical willingness to have their medications deprescribed, does not appear to reflect the actual changes in medication-related outcomes over time.

For peer review only

For peer review only - http://bmjopen.bmj.com/site/about/guidelines.xhtml

Introduction

Globally, there is increasing focus on polypharmacy in the older population. Up to 50% of older adults aged 65 years and above take one or more inappropriate medication,[1] which has been associated with harmful effects on health outcomes and quality of life.[2,3] In older patients with multiple chronic conditions (multimorbidity) the percentage is even higher.[4] A medication is considered *inappropriate* when potential harms outweigh potential benefits in the individual [5]. One strategy to mitigate against inappropriate medication use is deprescribing, the process of reducing or stopping medications that lack benefit or may cause harm.[6] However, implementing deprescribing decisions in clinical practice is challenging.

The extensive research into the barriers and facilitators of deprescribing has shown mixed results. Older adults often hold ambivalent attitudes in that they may express a willingness to reduce their medications whilst perceiving all their medications as beneficial and necessary.[7,8] Clinicians can perceive their patients are reluctant to have their medications deprescribed.[9,10] A recent study from Switzerland found a quarter of patients (22/87) declined their GP's offer to deprescribe a medication in a cluster-randomized study – even with a shared decision-making intervention.[11] Similarly, a substantial proportion of participants (30%-40%) decline to participate in deprescribing intervention studies.[12-15]

To understand patients' attitudes towards deprescribing, researchers have turned towards self-reported assessments such as the Patient Perceptions of Deprescribing survey[16] and the revised Patients' Attitudes Towards Deprescribing (rPATD) questionnaire.[17] The rPATD has high uptake in the deprescribing literature with the global question most frequently used: *"If my doctor said it was possible, I would be willing to stop one or more of my medicines"*. A systematic review of this questionnaire (and related versions) found inconsistency in whether there was statistical significance (and direction of the association) between characteristics and willingness to deprescribe[18]. However, mostly cross-sectional surveys were included, and few studies have used the rPATD in longitudinal research or investigating medication-related outcomes such as appropriateness or implementation of deprescribing.

For peer review only - http://bmjopen.bmj.com/site/about/guidelines.xhtml

It remains to be seen if patients' willingness to have medications deprescribed is associated with the implementation of actual deprescribing decisions and real changes in medication-related outcomes over time.

To address this gap in the deprescribing literature we aimed to investigate the association between older adults' willingness to have medications deprescribed and (1) actual change in their medications at 1-year follow-up, (2) change in the appropriateness of medications at 1-year follow-up, and (3) actual implementation of prescribing recommendations generated by an electronic decision support system tested in a clinical trial (OPTICA) to stop medications.

Methods

Overview of the OPTICA trial

The methods and results of the "Optimising PharmacoTherapy In the multimorbid elderly in primary Care" (OPTICA) trial (*clinicaltrials.gov identifier: NCT03724539*) have been reported elsewhere [19].[20-22] In brief, 323 patients from 43 GP practices were recruited into this cluster randomized clinical trial between January 2019 and February 2020. The 12-month follow-up ended in February 2021. 21 GPs with 160 patients were assigned to the intervention group and 22 GPs with 163 patients to the control group. Eligible patients were 65 years or older, they had ≥3 chronic conditions, and were taking ≥5 medications regularly. Baseline willingness to have medications deprescribed was assessed at baseline. While GPs in the control group continued to provide usual care to their patients including a discussion of patients' medications in accordance with their usual practice, GPs in the intervention group performed a structured medication review centred around an electronic clinical decision support system called the "Systematic Tool to Reduce Inappropriate Prescribing"-Assistant (STRIP-Assistant). This tool is based on the STOPP/START criteria and generated prescribing recommendations to stop, start, or adapt the dosage and flagged interactions.[23-25] The OPTICA trial had a pragmatic design with data collected from participants' electronic health records (e.g., medications, diagnoses) and from participants or their legal representatives over the phone (e.g., quality of life, living situation, etc.). The two primary outcomes of the trial were the improvement in the Medication Appropriateness Index (MAI) and the Assessment of Underutilization (AOU) at 12 months.[26-28] Secondary outcomes included the number of medications, number of falls and fractures

For peer review only - http://bmjopen.bmj.com/site/about/guidelines.xhtml

and quality of life. In the intention-to-treat analysis at 12 months, there were no group differences in the improvement of medication appropriateness (Odds ratio (OR)=1.05; 95% confidence interval (CI)=0.59 to 1.87) nor the number of prescribing omissions (OR=0.90; 95% CI=0.41 to 1.96). The per-protocol analysis showed no statistically significant group difference either and there were no group differences in the secondary outcomes. In 59% of participants at least one prescribing recommendation to stop or start a medication was implemented. It is of note that not all prescribing recommendations generated by STRIPA were accepted by GPs and discussed with patients. The OPTICA trial was approved by the Cantonal Ethics Committee of the Canton of Bern (*BASEC-ID: 2018–00914*). All participants or their legal representatives provided written informed consent.

Study design and Sample Definition

This is a longitudinal, post-hoc sub-study of data collected during the OPTICA trial. Data from the trial baseline, the 6-month follow-up, and the 12-month follow-up were used for the present analyses. This manuscript adheres to the Strengthening the Reporting of Observational Studies in Epidemiology (STROBE) reporting guideline for observational studies.[29] All 323 participants of the OPTICA trial were older adults (≥65 years of age), with multimorbidity (≥3 chronic conditions) and polypharmacy (≥5 medications). We limited the present analyses to the participants for whom the patient version of the 'revised Patient Attitudes Towards Deprescribing' (rPATD) was used (N = 298).[17]

Assessment of patients' willingness to have medications deprescribed

Patients' attitudes towards having medications deprescribed hypothetically was measured using the rPATD at baseline. The rPATD contains 22 questions with "Strongly disagree (1)" and "Strongly agree (5)" as the scale anchors.[17] For the main analyses, we used the global question from the rPATD "If my doctor said it was possible, I would be willing to stop one or more of my regular medicines" as the independent variable, which measures patients' willingness to accept deprescribing proposed by a medical doctor. In addition, we used two questions from the rPATD "appropriateness" factor ("I would like to try stopping one of my medicines to see how I feel without it" and "I would like my doctor to reduce the dose of one or more of my medicines"), which aim to

For peer review only - http://bmjopen.bmj.com/site/about/guidelines.xhtml

measure patients' desires to try to stop or reduce medicines (Box 1). Further, we used the rPATD "concerns about stopping" factor score (ranging from 1 to 5) calculated based on five rPATD questions as independent variables. Several questions from the rPATD were used as independent variables given the ceiling effect of the global rPATD willingness to deprescribe question and the fact that there is more variation in the responses to the other two rPATD questions and the concerns about stopping score.

Box 1: Attitudes towards deprescribing: rPATD questions

**Global question:**<br>*"If my doctor said it was possible, I would be willing to stop one or more of my regular medicines"*
**Appropriateness questions:**<br>*"I would like to try stopping one of my medicines to see how I feel without it"*<br>*"I would like my doctor to reduce the dose of one or more of my medicines"*
**Concerns about stopping questions:**<br>*"I have had a bad experience when stopping a medicine before"*<br>*"I would be reluctant to stop a medicine that I had been taking for a long time"*<br>*"If one of my medicines was stopped I would be worried about missing out on future benefits"*<br>*"I get stressed whenever changes are made to my medicines"*<br>*"If my doctor recommended stopping a medicine I would feel that he/she was giving up on me"*

Assessment of medication-related outcomes over time

Medication-related outcomes over time were assessed using data collected at baseline and throughout the follow-up period of the OPTICA trial. Details on how the three medication-related outcomes were assessed – change in the number of medications, medication appropriateness, and the implementation of prescribing recommendations – can be found in **Box 2** and in the published protocol.[20]

Box 2: Assessment of medication-related outcomes

	Outcome	Measurement
**Aim 1**	Number of long-term medications	Change in the number of long-term medications (≥90 days) between baseline and the 12-month follow-up.
**Aim 2**	Medication appropriateness	Change in the average medication appropriateness between baseline and the 12-month follow-up.<br>We first calculated the average MAI for the baseline and the 12-month follow-up by dividing the total MAI score of the respective timepoint by the number of long-term medications at this timepoint. Then we calculated the change in the average MAI between baseline and the 12-month follow-up.
**Aim 3**	Implementation of prescribing recommendations to stop medications	Binary variable describing whether any deprescribing recommendation to stop a medication generated by the electronic decision support system tested in the OPTICA

For peer review only - http://bmjopen.bmj.com/site/about/guidelines.xhtml

		trial had been implemented or not at the patient level. Only data from the OPTICA intervention group was used.

Co-variates

The following variables were used to adjust the analyses: gender, age, educational status, number of chronic conditions, living situation, capable of leaving the house (yes/no), patients' satisfaction with medications, and number of GP visits in the 6 months prior to the study enrolment.

Statistical analysis

First, we described the demographics and main clinical characteristics of the study participants. Second, we descriptively analysed three questions from the rPATD and the concerns about stopping score to describe patients' willingness to have medications deprescribed at baseline. Third, we performed a set of multilevel regression analyses. For Aims 1 and 2, we used multilevel linear regression models to investigate the association between patients' willingness to have medications deprescribed and the outcomes. In subgroup analyses, we restricted the analyses to the OPTICA intervention group. For Aim 3, we used a multilevel logistic regression model to investigate the association between patients' willingness to have medications deprescribed and the binary outcome variable. For Aim 3, we used data from the OPTICA intervention group only. All analyses were adjusted for the clustering effect at the GP level and the measurable co-variates listed in the section above plus the group allocation during the trial (except for the analyses for Aim 3, which were based on data from the intervention group only). Analyses were limited to the observed data and we did not use any multiple imputation methods. All analyses were performed with STATA 15.1 (StataCorp, College Station, TX, USA).

Results

Baseline characteristics

Table 1 describes the baseline characteristics of study participants**.** Out of the 298 participants for whom information on their willingness to have medications deprescribed was assessed at baseline (92% of all trial participants), 45% were women and the median age was 78 years. 75% (224/298) of the participants had equal or higher than median willingness to have medications deprescribed.

For peer review only - http://bmjopen.bmj.com/site/about/guidelines.xhtml

Table 1. Baseline characteristics of study participants by willingness to deprescribe (n = 298)

	All patients in the sample *(n=298)*[2]	Patients with lower than median willingness to deprescribe *(n=74)*[1]	Patients with equal or higher than median willingness to deprescribe *(n=224)*[1]
Age (in years)	78 (74-83)	79 (74-83)	78 (74-83)
Female	133 (45)	39 (53)	94 (42)
Patient education			
Mandatory schooling or less	113 (38)	25 (34)	86 (39)
Diploma at secondary school level	139 (47)	33 (45)	106 (47)
Higher education diploma	45 (15)	16 (22)	29 (13)
Number of chronic conditions	7 (5-10)	7 (5-9)	7 (5-11)
Living situation			
In apartment/ house without any external help	227 (76)	62 (84)	165 (74)
In apartment/ house with some external help	61 (20)	9 (12)	52 (23)
In a nursing home	10 (3)	3 (4)	7 (3)
Patient is unable to leave the house (as compared to not housebound)	7 (2)	2 (3)	5 (2)
Equal or higher than median satisfaction with current medication use (as compared to lower than medication willingness to deprescribe)	215 (72)	59 (80)	156 (70)
Number of GP consultations during the 6-month follow-up period prior to the enrolment into the study trial	11 (0-60)	9 (6-19)	11 (8-15)
Average Medication Appropriateness Index at baseline	3.10 (3.5)	3.6 (3.9)	2.4 (3.3)
Number of long-term medications	8.5 (6.1)	9.2 (4.6)	8.5 (5.9)
Group allocation during the trial	146 to control group, 152 to intervention group	36 to control group, 38 to intervention group	110 to control group, 114 to intervention group

For continuous variables the median and the interquartile range (IQR) are presented. For categorial variables frequencies and percentages are presented. | [1] Patients' willingness to have medications deprescribed was measured using the rPATD global question "If my doctor said it was possible, I would be willing to stop one or more of my regular medicines". The median willingness to have medications deprescribed corresponded to "strongly agree" with the rPATD global question. | [2] Among the 298 patients, 146 patients were then randomized to the control group and 152 patients to the intervention group. | Missingness: Gender, and age had 0% missing values. Patient education, living situation, housebound yes/no, patient satisfaction with medications, and the number of chronic medications had less than 3% missing information. The number of chronic conditions and the average Medication Appropriateness Index at baseline had less than 7% missing.

For peer review only - http://bmjopen.bmj.com/site/about/guidelines.xhtml

Proxy measures for patients' willingness to have medications deprescribed

Table 2 shows the descriptive results of the different measures used to assess patients' willingness to have medications deprescribed. More than 85% of participants strongly agreed or agreed with the rPATD global question and only 9% of participants disagreed with this statement, whereas there was slightly more variation in responses to the other two rPATD questions. Approximately 60% of participants reported they would like to try stopping one of their medications to see how they would feel without it, whereas 32% disagreed or strongly disagreed with this statement.

Table 2. Patients' attitudes towards having medications deprescribed[1] (n=298)

Name of the variable		*Frequency*	*Percent*
rPATD global question: "If my doctor said it was possible, I would be willing to stop one or more of my regular medicines"			
strongly disagree		13	4.4
disagree		14	4.7
unsure		9	3.0
agree		38	12.8
strongly agree		224	75.2
Alternative measurements of patients' willingness to have medications deprescribed based on the rPATD			
Concerns about stopping score	Mean (SD)	1.8 (0.8)	
	Median (IQR)	1.6 (1-2.4)	
'I would like to try stopping one of my medicines to see how I feel without it'			
strongly disagree		35	11.7
disagree		59	19.8
unsure		19	6.4
agree		65	21.8
strongly agree		120	40.3
'I would like my doctor to reduce the dose of one or more of my medicines'			
strongly disagree		28	9.4
disagree		29	9.7
unsure		24	8.1
agree		64	21.5
strongly agree		153	51.3

[1] As measured by the 'revised Patients' Attitudes Towards Deprescribing (rPATD) questionnaire. Source: Reeve, E., al. Development and Validation of the Revised Patients' Attitudes Towards Deprescribing (rPATD) Questionnaire: Versions for Older Adults and Caregivers. Drugs Aging 33, 913–928 (2016). | Missingness: There was 0% missingness in rPATD questions and the concerns of stopping score.

For peer review only

Number of medications over time

Table 3 shows the associations between the different measures assessing patients' willingness to have medications deprescribed and the change in number of medications throughout the 12-month follow-up period. The only statistically significant association was between the concerns about stopping score and the change in the number of medications (coefficient: 0.65, 95% CI: 0.08 to 1.22). A higher score indicates being more concerned about stopping. Meaning, per 1-unit increase in the concerns about stopping score the change in the number of medications between baseline and the

For peer review only - http://bmjopen.bmj.com/site/about/guidelines.xhtml

12-month follow-up increased by 0.65. In the sub-group analyses restricted to participants in the intervention group the results were identical (**eTable 1**).

Table 3. Multivariate associations between the change in the number of medications throughout the 12-month follow-up period and patients' willingness to deprescribe (n=253)

Name of the variable	*Coefficient*	*p-value*	*95% confidence interval*
rPATD global question: "If my doctor said it was possible, I would be willing to stop one or more of my regular medicines" (reference: strongly agree)			
agree	-0.96	0.169	-2.33 to 0.41
unsure	0.61	0.963	-2.52 to 2.64
disagree	0.58	0.598	-1.56 to 2.71
strongly disagree	0.26	0.806	-1.81 to 2.33
Alternative measurements of patients' willingness to have medications deprescribed based on the rPATD			
Concerns about stopping score (per 1-unit increase)[1]	0.65	**0.026**	0.08 to 1.22
'I would like to try stopping one of my medicines to see how I feel without it' (reference: strongly agree)			
agree	-0.12	0.830	-1.33 to 1.07
unsure	0.62	0.509	-1.24 to 2.51
disagree	0.47	0.448	-0.74 to 1.68
strongly disagree	-0.21	0.774	-1.68 to 1.25
'I would like my doctor to reduce the dose of one or more of my medicines' (reference: strongly agree)			
agree	1.13	0.070	-0.09 to 2.36
unsure	-0.97	0.251	-2.64 to 0.69
disagree	0.79	0.306	-0.72 to 2.31
strongly disagree	0.71	0.359	-0.81 to 2.24

Multilevel linear regression models adjusted for patient age, education status, gender, number of chronic conditions, living situation, whether the patient is housebound or not, patient satisfaction with medications, the number of GP consultations in the 6-months prior to the study inclusion, and the group allocation during the trial. | [1]As calculated based on Reeve, E., al. Development and Validation of the Revised Patients' Attitudes Towards Deprescribing (rPATD) Questionnaire: Versions for Older Adults and Caregivers. Drugs Aging 33, 913–928 (2016). | Missingness: The change in the number of chronic medications over the 12-month follow-up period had 8% missing values.

Medication appropriateness over time

The associations between patients' willingness to have medications deprescribed and the change in medication appropriateness throughout the 12-month follow-up period is shown in **Table 4**. We did not find evidence for the statistically significant associations. In the subgroup analyses restricted to the OPTICA intervention group, we found statistically significant associations between patients' being undecided or (strongly) agreeing with the statement "I would like my doctor to reduce the dose of one or more of my medicines" and an improvement in medication appropriateness between baseline and the 12-month follow-up period (results not presented).

Table 4. Multivariate associations between the change in the medication appropriateness[1] throughout the 12-month follow-up period and patients' willingness to deprescribe[2] (n=242)

Name of the variable	*Coefficient*	*p-value*	*95% confidence interval*
rPATD global question: "If my doctor said it was possible, I would be willing to stop one or more of my regular medicines" (reference: strongly agree)			
agree	0.35	0.426	-0.51 to 1.21

For peer review only - http://bmjopen.bmj.com/site/about/guidelines.xhtml

unsure	0.92	0.293	-0.79 to 2.63
disagree	-1.01	0.145	-2.36 to 0.35
strongly disagree	-0.80	0.221	-2.08 to 0.48
Alternative measurements of patients' willingness to have medications deprescribed based on the rPATD			
Concerns about stopping score (per 1-unit increase)[1]			
	-0.29	0.120	-0.65 to 0.08
'I would like to try stopping one of my medicines to see how I feel without it' (reference: strongly agree)			
agree	-0.45	0.253	-1.21 to 0.32
unsure	-0.66	0.281	-1.87 to 0.54
disagree	-0.45	0.246	-1.22 to 0.31
strongly disagree	-0.57	0.233	-1.51 to 0.37
'I would like my doctor to reduce the dose of one or more of my medicines' (reference: strongly agree)			
agree	-0.44	0.253	-1.20 to 0.32
unsure	-0.59	0.282	-1.67 to 0.49
disagree	-0.02	0.968	-0.95 to 0.99
strongly disagree	0.13	0.795	-0.85 to 1.11

Multilevel linear regression models adjusted for patient age, education status, gender, number of chronic conditions, living situation, whether the patient is housebound or not, patient satisfaction with medications, the number of GP consultations in the 6-months prior to the study inclusion, and the group allocation during the trial. | [1]As assessed using the Medication Appropriateness Index: Samsa GP, Hanlon JT, Schmader KE, Weinberger M, Clipp EC, Uttech KM, Lewis IK, Landsman PB, Cohen HJ. A summated score for the medication appropriateness index: development and assessment of clinimetric properties including content validity. J Clin Epidemiol. 1994 Aug;47(8):891-6. | [2]As calculated based on Reeve, E., al. Development and Validation of the Revised Patients' Attitudes Towards Deprescribing (rPATD) Questionnaire: Versions for Older Adults and Caregivers. Drugs Aging 33, 913–928 (2016). | Missingness: The change in the Medication Appropriateness Index over the 12-month follow-up period had 13% values.

Implementation of prescribing recommendations

Table 5 shows the association between patients' willingness to have medications deprescribed and the implementation of prescribing recommendations that were generated as part of the OPTICA medication review intervention. On average, 1 prescribing recommendation to stop or start a medication were reported to be implemented per patient (reported elsewhere[21]) and 59% of patients in the intervention group had 1 or more prescribing recommendation implemented. We did not find any evidence for a statistically significant association between patients' willingness to have medications deprescribed and the implementation of deprescribing recommendations.

Table 5. Multivariate associations between the implementation of recommendations to stop medications and patients' willingness to deprescribe[1] (n=31)

Name of the variable	*Odds ratio*	*p-value*	*95% confidence interval*
rPATD global question: "If my doctor said it was possible, I would be willing to stop one or more of my regular medicines" (reference: equal or higher than median agreement)[2]			
Below median agreement	4.90	0.244	0.34 to 71.3
Alternative measurements of patients' willingness to have medications deprescribed based on the rPATD			
Concerns about stopping score (per 1-unit increase)			
	1.13	0.812	0.41 to 3.13
'I would like to try stopping one of my medicines to see how I feel without it' (reference: equal or higher than median agreement)[2]			
Below median agreement	2.53	0.305	0.43 to 14.89
'I would like my doctor to reduce the dose of one or more of my medicines' (reference: equal or higher than median agreement)[2]			
Below median agreement	7.8	0.086	0.75 to 82.2

For peer review only - http://bmjopen.bmj.com/site/about/guidelines.xhtml

[1] Multilevel logistic regression models adjusted for patient age, and gender. | [2] Due to the low number of observations for which the implementation of recommendations was reported, the rPATD question was dichotomized.
The analyses presented in this table used data from the OPTICA intervention group only. Despite several reminders, only a couple of general practitioners from the OPTCIA intervention group reported this information.

Discussion

In this sub-study of a cluster randomised clinical trial, we examined the association between older adults' hypothetical willingness to have one or more medications deprescribed and change in a participant's medications, appropriateness of their medications, and actual implementation of prescribing recommendations. Overall, we found that these medication-related outcomes measured over time were not associated with the rPATD deprescribing questions measured in this study. To consider reasons why no association was found, firstly we discuss the rPATD questions in more detail and their ability to measure self-reported attitudes towards deprescribing. Secondly, consideration is given to our deprescribing intervention and how medication-related outcomes were measured in this study.

In our study, 88% of participants agreed or strongly agreed with the rPATD global question: "If my doctor said it was possible, I would be willing to have one or more of my medications deprescribed". However, this high agreement was not associated with changes in medication-related outcomes over time. This finding is in line with other deprescribing intervention trials using the rPATD.[30-32] These studies also reported high agreement with hypothetical willingness to deprescribe (86-95%) but found no effect on deprescribing or medication-related outcomes – except for one. This study found that a higher willingness to deprescribe was not only associated with a higher rate of deprescribing but also initiating medicines.[18] The majority of studies using the rPATD global deprescribing question report greater than 80% agreement with hypothetical willingness to deprescribe.[8] Therefore, it may be difficult to find an association with the global question and deprescribing or medication-related outcomes given the ceiling effect and social desirability bias of the question.

The global rPATD question could be measuring a person's agreement with the doctor rather than deprescribing as there is substantial variation between it and other rPATD deprescribing questions which do not refer to the doctor.[8] In our study participants' responses were much higher for the rPATD global deprescribing question with 88% of participants willing to deprescribe hypothetically if their doctor said it was possible, however 62% wanted to try stopping one of their medications to

For peer review only - http://bmjopen.bmj.com/site/about/guidelines.xhtml

see how they would feel without it. Other studies using the rPATD frequently report substantial differences between these two questions. In 15 studies using the rPATD (10 from a systematic review[8] and 5 published subsequently[33-37]) there were none with less than a 30% gap between these questions – with the global question responses always higher (up to 73% difference). This indicates that depending on which question is used to assess patients' willingness to deprescribe from the rPATD, patients may be misclassified.

Additionally, the connection between an older adult's attitudes towards medications and their desire to follow their doctor's recommendations should not be underestimated. A content analysis[38] including over 2,500 participants from Australia, the UK and the US found that approximately one-half of older adults who agreed with deprescribing (in a hypothetical scenario) felt that the doctor's recommendation was an important consideration.

There is a complex interplay of factors, such as clinical decision-making and patients' attitudes, that are behind deprescribing. From our study, we cannot say why recommendations of medication changes were or were not implemented. This is a common problem in deprescribing trials – and in clinical practice to a certain extent – where deprescribing has not occurred and we do not know why. There is a need to capture the reasons behind patients or doctors resisting deprescribing in interventional and survey studies, as this is a gap in deprescribing research.

Future work should look towards developing and using attitudinal measures that are less vulnerable to the influence of the doctor and that are sensitive to reluctance towards deprescribing. Additionally, it may be helpful to capture attitudes towards deprescribing for specific medications.[37,39] Ultimately, it would be useful for a tool to reliably identify patients at different degrees of willingness to deprescribe so that deprescribing interventions can be tailored to their needs and preferences.

Strengths and Limitations

The present analyses were strengthened by the longitudinal design, which allows for a clear temporal distinction between patients' willingness to have medications deprescribed assessed at baseline and the medication-related outcomes over time in addition to offer the intervention to optimize medication randomly. We would like to emphasize the following limitations of these present analyses. Firstly, patients agreeing to participate in the OPTICA trial could have had a higher willingness to have one or more of their medications deprescribed. Due to challenges with how data from the electronic

For peer review only - http://bmjopen.bmj.com/site/about/guidelines.xhtml

health records of participating patients were recorded, there was some missing data on medication, which is why some participants were excluded from the analyses. Also, to determine the medication-related outcomes for aim 1 and 2 we used prescribing data from electronic health records, which does not necessarily correspond to what medications were actually used by patients. Finally, despite several reminders, only a small proportion of GPs from the intervention group reported which and how many prescribing recommendations were implemented together with patients. This explains the smaller sample size for our third aim.

Conclusions

Our findings indicate that there is no association between patients' willingness to have medications deprescribed and medication-related outcomes over time. It is important to capture a range of participant attitudes that are both for and against deprescribing, as well as to consider the relationship between self-report surveys and actual deprescribing. The results highlight the need for further research to better understand the factors that contribute to successful deprescribing in primary care settings.

For peer review only

Declarations

Data sharing statement

We will make the data for this study available to other researchers upon request after publication. The data will be made available for scientific research purposes, after the proposed analysis plan has been approved. Data and documentation will be made available through a secure file exchange platform after approval of the proposal. In addition, a data transfer agreement must be signed (which defines obligations that the data requester must adhere to with regard to privacy and data handling). Deidentified participant data limited to the data used for the proposed project will be made available, along with a data dictionary and annotated case report forms. For data access, please contact the corresponding author.

For peer review only

Ethical approval

The study protocol of the OPTICA trial and other documentation was approved by the competent ethics committee of the canton of Bern (KEK), Switzerland, and the Swiss regulatory authority (Swissmedic) (BASEC ID: 2018–00914). The KEK and Swissmedic received annually safety reports and were informed about the end of the study. All participants gave their written informed consent. The OPTICA trial was performed in accordance with relevant regulations and guidelines.

Funding

The OPTICA trial was supported by the Swiss National Science Foundation, within the framework of the National Research Programme 74 "Smarter Health Care" (NRP74) under contract number 407440_167465 (to Prof Streit, Prof Rodondi, and Prof Schwenkglenks). Dr Jungo was funded by a Postdoc.Mobility Fellowship from the Swiss National Science Foundation (P500PM_206728). Dr Weir was funded by a Swiss Government Excellence Scholarship and a Swiss National Science Foundation Scientific Exchanges grant.

Role of the Funder

The funders had no role in the design and conduct of the study; collection, management, analysis, and interpretation of the data; preparation, review, or approval of the manuscript; and decision to submit the manuscript for publication.

Conflict of interest disclosures

The authors do not have any conflicts of interest to disclose.

Acknowledgements

We would like to thank the GPs who participated in the OPTICA trial for their great contributions without which it would have been impossible to conduct this study. We also thank the participants for consenting to participate in our study. Katharina Tabea Jungo is a member of the Junior Investigator Intensive Program of the US Deprescribing Research Network, which is funded by the National Institute on Aging.

Author contributions

Dr Jungo and Dr Weir had full access to all the data in the study and takes responsibility for the integrity of the data and the accuracy of the data analysis.

Concept and design: All authors.

Acquisition, analysis, or interpretation of data: All authors.

Drafting of the manuscript: Weir and Jungo.

Critical revision of the manuscript for important intellectual content: All authors.

Statistical analysis: Jungo

Obtained funding: Streit.

Administrative, technical, or material support: Jungo, Streit.

Supervision: Streit.

For peer review only - http://bmjopen.bmj.com/site/about/guidelines.xhtml

References

1. Maher RL, Hanlon J, Hajjar ER. Clinical consequences of polypharmacy in elderly. *Expert Opinion on Drug Safety*. 2014/01/01 2014;13(1):57-65. doi:10.1517/14740338.2013.827660
2. Fried TR, O'Leary J, Towle V, Goldstein MK, Trentalange M, Martin DK. Health outcomes associated with polypharmacy in community-dwelling older adults: a systematic review. *J Am Geriatr Soc*. Dec 2014;62(12):2261-72. doi:10.1111/jgs.13153
3. Kantor ED, Rehm CD, Haas JS, Chan AT, Giovannucci EL. Trends in Prescription Drug Use Among Adults in the United States From 1999-2012. *Jama*. Nov 3 2015;314(17):1818-31. doi:10.1001/jama.2015.13766
4. Jungo KT, Streit S, Lauffenburger JC. Utilization and Spending on Potentially Inappropriate Medications by US Older Adults with Multiple Chronic Conditions using Multiple Medications. *Archives of Gerontology and Geriatrics*. 2021/03/01/ 2021;93:104326. doi:https://doi.org/10.1016/j.archger.2020.104326
5. American Geriatrics Society 2019 Updated AGS Beers Criteria® for Potentially Inappropriate Medication Use in Older Adults. *J Am Geriatr Soc*. Apr 2019;67(4):674-694. doi:10.1111/jgs.15767
6. Scott IA, Hilmer SN, Reeve E, et al. Reducing Inappropriate Polypharmacy: The Process of DeprescribingReducing Inappropriate PolypharmacyReducing Inappropriate Polypharmacy. *JAMA Internal Medicine*. 2015;175(5):827-834. doi:10.1001/jamainternmed.2015.0324
7. Weir K, Nickel B, Naganathan V, et al. Decision-Making Preferences and Deprescribing: Perspectives of Older Adults and Companions About Their Medicines. *J Gerontol B Psychol Sci Soc Sci*. Sep 20 2018;73(7):e98-e107. doi:10.1093/geronb/gbx138
8. Weir KR, Ailabouni NJ, Schneider CR, Hilmer SN, Reeve E. Consumer attitudes towards deprescribing: a systematic review and meta-analysis. *The journals of gerontology Series A, Biological sciences and medical sciences*. Aug 14 2021;doi:10.1093/gerona/glab222
9. Weir KR, Naganathan V, Carter SM, et al. The role of older patients' goals in GP decision-making about medicines: a qualitative study. *BMC Family Practice*. 2021/01/08 2021;22(1):13. doi:10.1186/s12875-020-01347-y
10. Ailabouni NJ, Nishtala PS, Mangin D, Tordoff JM. General practitioners' insight into deprescribing for the multimorbid older individual: a qualitative study. *International Journal of Clinical Practice*. 2016;70(3):261-276. doi:https://doi.org/10.1111/ijcp.12780
11. Zechmann S, Senn O, Valeri F, et al. Effect of a patient-centred deprescribing procedure in older multimorbid patients in Swiss primary care - A cluster-randomised clinical trial. *BMC geriatrics*. 2020/11/16 2020;20(1):471. doi:10.1186/s12877-020-01870-8
12. Tannenbaum C, Martin P, Tamblyn R, Benedetti A, Ahmed S. Reduction of Inappropriate Benzodiazepine Prescriptions Among Older Adults Through Direct Patient Education: The EMPOWER Cluster Randomized Trial. *JAMA Internal Medicine*. 2014;174(6):890-898. doi:10.1001/jamainternmed.2014.949
13. Beer C, Loh PK, Peng YG, Potter K, Millar A. A pilot randomized controlled trial of deprescribing. *Therapeutic advances in drug safety*. Apr 2011;2(2):37-43. doi:10.1177/2042098611400332
14. Potter K, Flicker L, Page A, Etherton-Beer C. Deprescribing in frail older people: a randomised controlled trial. *PloS one*. 2016;11doi:10.1371/journal.pone.0149984
15. Komagamine J, Sugawara K, Hagane K. Characteristics of elderly patients with polypharmacy who refuse to participate in an in-hospital deprescribing intervention: a retrospective cross-sectional study. *BMC geriatrics*. Apr 17 2018;18(1):96. doi:10.1186/s12877-018-0788-1
16. Linsky AM, Stolzmann K, Meterko M. The Patient Perceptions of Deprescribing (PPoD) Survey: Short-Form Development. *Drugs Aging*. Dec 2020;37(12):909-916. doi:10.1007/s40266-020-00802-w
17. Reeve E, Low LF, Shakib S, Hilmer SN. Development and Validation of the Revised Patients' Attitudes Towards Deprescribing (rPATD) Questionnaire: Versions for Older Adults and Caregivers. *Drugs Aging*. Dec 2016;33(12):913-928. doi:10.1007/s40266-016-0410-1
18. McCarthy C, Flood M, Clyne B, et al. *Association between patient attitudes towards deprescribing and subsequent prescription changes (preprint)*. 2023. https://www.authorea.com/doi/full/10.22541/au.167407899.90617773

For peer review only - http://bmjopen.bmj.com/site/about/guidelines.xhtml

19. Jungo KT, Ansorg A-K, Floriani C, et al. Optimizing Prescribing in Older Adults with Multimorbidity and Polypharmacy in Primary Care: A Cluster Randomized Clinical Trial (OPTICA Trial). *medRxiv*. 2022:2022.10. 31.22281164.
20. Jungo KT, Rozsnyai Z, Mantelli S, et al. 'Optimising PharmacoTherapy In the multimorbid elderly in primary CAre' (OPTICA) to improve medication appropriateness: study protocol of a cluster randomised controlled trial. *BMJ Open*. 2019;9(9):e031080. doi:10.1136/bmjopen-2019-031080
21. Jungo KT, Ansorg A-K, Floriani C, et al. Optimizing Prescribing in Older Adults with Multimorbidity and Polypharmacy in Primary Care: A Cluster Randomized Clinical Trial (OPTICA Trial). *medRxiv*. 2022:2022.10.31.22281164. doi:10.1101/2022.10.31.22281164
22. Jungo KT, Meier R, Valeri F, et al. Baseline characteristics and comparability of older multimorbid patients with polypharmacy and general practitioners participating in a randomized controlled primary care trial. *BMC Family Practice*. 2021/06/22 2021;22(1):123. doi:10.1186/s12875-021-01488-8
23. Drenth-van Maanen AC, Leendertse AJ, Jansen PAF, et al. The Systematic Tool to Reduce Inappropriate Prescribing (STRIP): Combining implicit and explicit prescribing tools to improve appropriate prescribing. *J Eval Clin Pract*. 2018;24(2):317-322. doi:https://dx.doi.org/10.1111/jep.12787
24. Meulendijk MC, Spruit MR, Drenth-van Maanen AC, et al. Computerized Decision Support Improves Medication Review Effectiveness: An Experiment Evaluating the STRIP Assistant's Usability. journal article. *Drugs & Aging*. June 01 2015;32(6):495-503. doi:10.1007/s40266-015-0270-0
25. Meulendijk MC, Spruit MR, Willeboordse F, et al. Efficiency of Clinical Decision Support Systems Improves with Experience. *Journal of medical systems*. Apr 2016;40(4):76. doi:10.1007/s10916-015-0423-z
26. Hanlon JT, Schmader KE, Ruby CM, Weinberger M. Suboptimal prescribing in older inpatients and outpatients. *J Am Geriatr Soc*. Feb 2001;49(2):200-9.
27. Hanlon JT, Schmader KE, Samsa GP, et al. A method for assessing drug therapy appropriateness. *Journal of clinical epidemiology*. Oct 1992;45(10):1045-51.
28. Samsa GP, Hanlon JT, Schmader KE, et al. A summated score for the medication appropriateness index: development and assessment of clinimetric properties including content validity. *Journal of clinical epidemiology*. Aug 1994;47(8):891-6.
29. von Elm E, Altman DG, Egger M, Pocock SJ, Gøtzsche PC, Vandenbroucke JP. The Strengthening the Reporting of Observational Studies in Epidemiology (STROBE) statement: guidelines for reporting observational studies. *Journal of clinical epidemiology*. Apr 2008;61(4):344-9. doi:10.1016/j.jclinepi.2007.11.008
30. Kouladjian O'Donnell L, Gnjidic D, Sawan M, et al. Impact of the Goal-directed Medication Review Electronic Decision Support System on Drug Burden Index: A cluster-randomised clinical trial in primary care. *Br J Clin Pharmacol*. Sep 22 2020;doi:10.1111/bcp.14557
31. Anderson K, Freeman C, Foster M, Scott I. GP-Led Deprescribing in Community-Living Older Australians: An Exploratory Controlled Trial. *J Am Geriatr Soc*. Feb 2020;68(2):403-410. doi:10.1111/jgs.16273
32. Turner JP, Martin P, Zhang YZ, Tannenbaum C. Patients beliefs and attitudes towards deprescribing: Can deprescribing success be predicted? *Research in social & administrative pharmacy : RSAP*. Apr 2020;16(4):599-604. doi:10.1016/j.sapharm.2019.07.007
33. Rozsnyai Z, Jungo KT, Reeve E, et al. What do older adults with multimorbidity and polypharmacy think about deprescribing? The LESS study - a primary care-based survey. *BMC geriatrics*. Oct 31 2020;20(1):435. doi:10.1186/s12877-020-01843-x
34. Navid P, Nguyen L, Jaber D, et al. Attitudes toward deprescribing among adults with heart failure with preserved ejection fraction. *J Am Geriatr Soc*. Jul 2021;69(7):1948-1955. doi:10.1111/jgs.17204
35. Crutzen S, Abou J, Smits SE, et al. Older people's attitudes towards deprescribing cardiometabolic medication. *BMC geriatrics*. Jun 16 2021;21(1):366. doi:10.1186/s12877-021-02249-z
36. Lukacena KM, Keck JW, Freeman PR, Harrington NG, Huffmyer MJ, Moga DC. Patients' attitudes toward deprescribing and their experiences communicating with clinicians and pharmacists. *Therapeutic advances in drug safety*. 2022;13:20420986221116465. doi:10.1177/20420986221116465
37. Bužančić I, Dragović P, Pejaković TI, Markulin L, Ortner-Hadžiabdić M. Exploring Patients' Attitudes Toward Deprescribing and Their Perception of Pharmacist Involvement in a European Country: A Cross-Sectional Study. *Patient preference and adherence*. 2021;15:2197-2208. doi:10.2147/ppa.S323846

For peer review only - http://bmjopen.bmj.com/site/about/guidelines.xhtml

38. Vordenberg SE, Rana R, Shang J, Choi J, Scherer AM, Weir KR. Reasons why older adults in three countries agreed with a deprescribing recommendation in a hypothetical vignette. *Basic & clinical pharmacology & toxicology*. 2023;

39. Lüthold RV, Jungo KT, Weir KR, et al. Understanding older patients' willingness to have medications deprescribed in primary care: a protocol for a cross-sectional survey study in nine European countries. *BMC geriatrics*. 2022/11/30 2022;22(1):920. doi:10.1186/s12877-022-03562-x

For peer review only

For peer review only - http://bmjopen.bmj.com/site/about/guidelines.xhtml

STROBE Statement—checklist of items that should be included in reports of observational studies

	Item No	Recommendation
**Title and abstract**	1	(*a*) Indicate the study's design with a commonly used term in the title or the abstract<br>✓ Page 2
		(*b*) Provide in the abstract an informative and balanced summary of what was done and what was found<br>✓ Page 2
**Introduction**		
Background/rationale	2	Explain the scientific background and rationale for the investigation being reported ✓ Page 4
Objectives	3	State specific objectives, including any prespecified hypotheses
**Methods**		
Study design	4	Present key elements of study design early in the paper<br>✓ Page 5
Setting	5	Describe the setting, locations, and relevant dates, including periods of recruitment, exposure, follow-up, and data collection<br>✓ Page 5-6
Participants	6	(*a*) *Cohort study*—Give the eligibility criteria, and the sources and methods of selection of participants. Describe methods of follow-up<br>*Case-control study*—Give the eligibility criteria, and the sources and methods of case ascertainment and control selection. Give the rationale for the choice of cases and controls<br>*Cross-sectional study*—Give the eligibility criteria, and the sources and methods of selection of participants
		(*b*) *Cohort study*—For matched studies, give matching criteria and number of exposed and unexposed<br>*Case-control study*—For matched studies, give matching criteria and the number of controls per case
Variables	7	Clearly define all outcomes, exposures, predictors, potential confounders, and effect modifiers. Give diagnostic criteria, if applicable<br>✓ Page 7-8
Data sources/ measurement	8*	For each variable of interest, give sources of data and details of methods of assessment (measurement). Describe comparability of assessment methods if there is more than one group<br>✓ Page 7-8
Bias	9	Describe any efforts to address potential sources of bias<br>✓ Page 14
Study size	10	Explain how the study size was arrived at<br>✓ Page 6
Quantitative variables	11	Explain how quantitative variables were handled in the analyses. If applicable, describe which groupings were chosen and why<br>✓ Page 8
Statistical methods	12	(*a*) Describe all statistical methods, including those used to control for confounding

For peer review only

		✓ Page 8
		(*b*) Describe any methods used to examine subgroups and interactions<br>✓ Page 8
		(*c*) Explain how missing data were addressed<br>✓ Page 8 + legend of Table 1
		(*d*) *Cohort study*—If applicable, explain how loss to follow-up was addressed<br>*Case-control study*—If applicable, explain how matching of cases and controls was addressed<br>*Cross-sectional study*—If applicable, describe analytical methods taking account of sampling strategy
		(*e*) Describe any sensitivity analyses

Continued on next page

**Results**		
Participants	13*	(a) Report numbers of individuals at each stage of study—eg numbers potentially eligible, examined for eligibility, confirmed eligible, included in the study, completing follow-up, and analysed<br>✓ Page 6
		(b) Give reasons for non-participation at each stage
		(c) Consider use of a flow diagram
Descriptive data	14*	(a) Give characteristics of study participants (eg demographic, clinical, social) and information on exposures and potential confounders<br>✓ Table 1
		(b) Indicate number of participants with missing data for each variable of interest<br>✓ Legend of Table 1
		(c) *Cohort study*—Summarise follow-up time (eg, average and total amount)
Outcome data	15*	*Cohort study*—Report numbers of outcome events or summary measures over time
		Case-control study—Report numbers in each exposure category, or summary measures of exposure
		Cross-sectional study—Report numbers of outcome events or summary measures
Main results	16	(*a*) Give unadjusted estimates and, if applicable, confounder-adjusted estimates and their precision (eg, 95% confidence interval). Make clear which confounders were adjusted for and why they were included<br>Unadjusted estimates are available upon request.
		(*b*) Report category boundaries when continuous variables were categorized<br>✓ Page 9
		(*c*) If relevant, consider translating estimates of relative risk into absolute risk for a meaningful time period
Other analyses	17	Report other analyses done—eg analyses of subgroups and interactions, and sensitivity analyses
**Discussion**		
Key results	18	Summarise key results with reference to study objectives<br>✓ Page 12-13
Limitations	19	Discuss limitations of the study, taking into account sources of potential bias or imprecision. Discuss both direction and magnitude of any potential bias<br>✓ Page 14
Interpretation	20	Give a cautious overall interpretation of results considering objectives, limitations, multiplicity of analyses, results from similar studies, and other relevant evidence

For peer review only - http://bmjopen.bmj.com/site/about/guidelines.xhtml

		✓ Page 12-14
Generalisability	21	Discuss the generalisability (external validity) of the study results<br>✓ Page 14
**Other information**		
Funding	22	Give the source of funding and the role of the funders for the present study and, if applicable, for the original study on which the present article is based<br>✓ Page 16

*Give information separately for cases and controls in case-control studies and, if applicable, for exposed and unexposed groups in cohort and cross-sectional studies.

Note: An Explanation and Elaboration article discusses each checklist item and gives methodological background and published examples of transparent reporting. The STROBE checklist is best used in conjunction with this article (freely available on the Web sites of PLoS Medicine at http://www.plosmedicine.org/, Annals of Internal Medicine at http://www.annals.org/, and Epidemiology at http://www.epidem.com/). Information on the STROBE Initiative is available at www.strobe-statement.org.

For peer review only

BMJ Open

Older adults' willingness to deprescribe and medication changes: A longitudinal sub-study of a cluster randomized controlled trial

Journal:	*BMJ Open*
Manuscript ID	bmjopen-2023-075325.R1
Article Type:	Original research
Date Submitted by the Author:	07-Sep-2023
Complete List of Authors:	Jungo, Katharina Tabea; University of Bern<br>Weir, Kristie; University of Bern; The University of Sydney<br>Cateau, Damien; University of Geneva, Institute of Pharmaceutical Sciences of Western Switzerland; University of Lausanne, Community Pharmacy, Center for Primary Care and Public Health (Unisanté)<br>Streit, Sven; University of Bern
**Primary Subject Heading**:	General practice / Family practice
Secondary Subject Heading:	General practice / Family practice, Health services research
Keywords:	Aged, Primary Health Care, Primary Care < Primary Health Care

SCHOLARONE™
Manuscripts

I, the Submitting Author has the right to grant and does grant on behalf of all authors of the Work (as defined in the below author licence), an exclusive licence and/or a non-exclusive licence for contributions from authors who are: i) UK Crown employees; ii) where BMJ has agreed a CC-BY licence shall apply, and/or iii) in accordance with the terms applicable for US Federal Government officers or employees acting as part of their official duties; on a worldwide, perpetual, irrevocable, royalty-free basis to BMJ Publishing Group Ltd ("BMJ") its licensees and where the relevant Journal is co-owned by BMJ to the co-owners of the Journal, to publish the Work in this journal and any other BMJ products and to exploit all rights, as set out in our licence.

The Submitting Author accepts and understands that any supply made under these terms is made by BMJ to the Submitting Author unless you are acting as an employee on behalf of your employer or a postgraduate student of an affiliated institution which is paying any applicable article publishing charge ("APC") for Open Access articles. Where the Submitting Author wishes to make the Work available on an Open Access basis (and intends to pay the relevant APC), the terms of reuse of such Open Access shall be governed by a Creative Commons licence – details of these licences and which Creative Commons licence will apply to this Work are set out in our licence referred to above.

Other than as permitted in any relevant BMJ Author's Self Archiving Policies, I confirm this Work has not been accepted for publication elsewhere, is not being considered for publication elsewhere and does not duplicate material already published. I confirm all authors consent to publication of this Work and authorise the granting of this licence.

For peer review only

Older adults' willingness to deprescribe and medication changes: A longitudinal sub-study of a cluster randomized controlled trial

Author list: Katharina Tabea Jungo* [1], PhD, Kristie Rebecca Weir* [1,2], PhD, Damien Cateau [3,4], PhD, Sven Streit [1], PhD
*shared first

Affiliations
[1] Institute of Primary Health Care BIHAM, University of Bern, Bern, Switzerland
[2] Sydney School of Public Health, Faculty of Medicine and Health, The University of Sydney, Sydney, Australia
[3] Community Pharmacy, Centre for Primary Care and Public Health (Unisanté), University of Lausanne, Lausanne, Switzerland
[4] School of Pharmaceutical Sciences, University of Geneva, Geneva, Switzerland

Corresponding author:
Kristie Rebecca Weir
kristie.weir@unibe.ch
Mittelstrasse 43, 3012 Bern, Institute of Primary Health Care BIHAM, University of Bern, Bern, Switzerland

Word count manuscript: 2,930

Key words (5): polypharmacy, deprescribing, older adults, comorbidity, medications

For peer review only - http://bmjopen.bmj.com/site/about/guidelines.xhtml

Abstract

Objective: To investigate the association between older patients' willingness to have one or more medications deprescribed and: (1) change in medications, (2) change in the appropriateness of medications, and (3) implementation of prescribing recommendations generated by the electronic decision support system tested in the 'Optimizing PharmacoTherapy In the Multimorbid Elderly in Primary CAre' (OPTICA) trial.

Design: A longitudinal sub-study of the OPTICA trial, a cluster randomized controlled trial.

Setting: Swiss primary care settings.

Participants: Participants were aged ≥65 years, with ≥3 chronic conditions, and ≥5 regular medications recruited from 43 GP practices.

Exposures: Patients' willingness to have medications deprescribed was assessed using 3 questions from the 'revised Patient Attitudes Towards Deprescribing' (rPATD) questionnaire and its concerns about stopping score.

Measures/Analyses: Medication-related outcomes were collected at 1-year follow-up. Aim 1 outcome: change in the number of long-term medications between baseline and 12-month follow-up. Aim 2 outcome: change in medication appropriateness (Medication Appropriateness Index). Aim 3 outcome: binary variable on whether any prescribing recommendation generated during the OPTICA medication review was implemented. We used multilevel linear regression analyses (Aim 1, Aim 2) and multilevel logistic regression analyses (Aim 3). Models were adjusted for sociodemographic variables and the clustering effect at GP level.

Results: 298 patients completed the rPATD, 45% were women and 78 was the median age. A statistically significant association was found between the concerns about stopping score and the change in the number of medications over time (per 1-unit increase in the score the average number of medications use was 0.65 higher; 95% CI: 0.08 to 1.22). There was no statistically significant association between patients' willingness to have medications deprescribed and medication-related outcomes.

Conclusions: These findings indicate that there is no association between patients' willingness to have medications deprescribed and medication-related outcomes over one year.

Trial registration: NCT03724539Clinicaltrials.gov NCT03724539

For peer review only - http://bmjopen.bmj.com/site/about/guidelines.xhtml

Strengths and limitations

- A longitudinal sub-study (n=298 patients) of the OPTICA trial – a cluster randomized controlled trial conducted in Swiss primary care settings.
- A self-reported questionnaire used to measure patients' hypothetical willingness to have their medications deprescribed and medication-related outcomes after 1 year was investigated.
- The medication-related outcomes were the number of medications between baseline and 12-month follow-up, and medication appropriateness (as measured by the Medication Appropriateness Index).
- The longitudinal study design allowed for a clear temporal distinction between patients' willingness to have medications deprescribed assessed at baseline and the medication-related outcomes after 1 year.
- Older adults agreeing to participate in the OPTICA trial could have had a higher willingness to have one or more of their medications deprescribed.

For peer review only - http://bmjopen.bmj.com/site/about/guidelines.xhtml

Introduction

Globally, there is increasing focus on polypharmacy in the older population. Up to 50% of older adults aged 65 years and above take one or more inappropriate medication,[1] which has been associated with harmful effects on health outcomes and quality of life.[2, 3] In older patients with multiple chronic conditions (multimorbidity) the percentage is even higher.[4] A medication is considered *inappropriate* when potential harms outweigh potential benefits in the individual [5]. One strategy to mitigate against inappropriate medication use is deprescribing, the process of reducing or stopping medications that lack benefit or may cause harm.[6] However, implementing deprescribing decisions in clinical practice is challenging.

The extensive research into the barriers and facilitators of deprescribing has shown mixed results. Older adults often hold ambivalent attitudes in that they may express a willingness to reduce their medications whilst perceiving all their medications as beneficial and necessary.[7, 8] Clinicians can perceive their patients are reluctant to have their medications deprescribed.[9, 10] A recent study from Switzerland found a quarter of patients (22/87) declined their GP's offer to deprescribe a medication in a cluster-randomized study – even with a shared decision-making intervention.[11] Similarly, a substantial proportion of participants (42% - 75%) decline to participate in deprescribing intervention studies.[12-14]

To understand patients' attitudes towards deprescribing, researchers have turned towards self-reported assessments such as the Patient Perceptions of Deprescribing survey [15] and the revised Patients' Attitudes Towards Deprescribing (rPATD) questionnaire.[16] The rPATD has high uptake in the deprescribing literature with the global question most frequently used: *"If my doctor said it was possible, I would be willing to stop one or more of my medicines"*. A systematic review of this questionnaire (and related versions) found inconsistency in whether there was statistical significance (and direction of the association) between characteristics and willingness to deprescribe.[17] However, mostly cross-sectional surveys were included, and few studies have used the rPATD in longitudinal research or investigating medication-related outcomes such as appropriateness or implementation of deprescribing.

For peer review only - http://bmjopen.bmj.com/site/about/guidelines.xhtml

It remains to be seen if patients' willingness to have medications deprescribed is associated with the implementation of actual deprescribing decisions and real changes in medication-related outcomes over time.

To address this gap in the deprescribing literature we aimed to investigate the association between older adults' willingness to have medications deprescribed and (1) actual change in their medications at 1-year follow-up, (2) change in the appropriateness of medications at 1-year follow-up, and (3) actual implementation of prescribing recommendations generated by an electronic decision support system tested in a clinical trial (OPTICA) to stop medications.

Methods

Overview of the OPTICA trial

The methods and results of the "Optimising PharmacoTherapy In the multimorbid elderly in primary Care" (OPTICA) trial (*clinicaltrials.gov identifier: NCT03724539*) have been reported elsewhere.[18-20] In brief, 323 patients from 43 GP practices were recruited into this cluster randomized clinical trial between January 2019 and February 2020. The 12-month follow-up ended in February 2021. 21 GPs with 160 patients were assigned to the intervention group and 22 GPs with 163 patients to the control group. Eligible patients were 65 years or older, they had ≥3 chronic conditions, and were taking ≥5 medications regularly. Baseline willingness to have medications deprescribed was assessed at baseline. While GPs in the control group continued to provide usual care to their patients including a discussion of patients' medications in accordance with their usual practice, GPs in the intervention group performed a structured medication review centred around an electronic clinical decision support system called the "Systematic Tool to Reduce Inappropriate Prescribing"-Assistant (STRIP-Assistant). This tool is based on the STOPP/START criteria and generated prescribing recommendations to stop, start, or adapt the dosage and flagged interactions.[21-23] The OPTICA trial had a pragmatic design with data collected from participants' electronic health records (e.g., medications, diagnoses) and from participants or their legal representatives over the phone (e.g., quality of life, living situation, etc.). The two primary outcomes of the trial were the improvement in the Medication Appropriateness Index (MAI) and the Assessment of Underutilization (AOU) at 12 months.[24-26] Secondary outcomes included the number of medications, number of falls and

For peer review only - http://bmjopen.bmj.com/site/about/guidelines.xhtml

fractures and quality of life. In the intention-to-treat analysis at 12 months, there were no group differences in the improvement of medication appropriateness (Odds ratio (OR)=1.05; 95% confidence interval (CI)=0.59 to 1.87) nor the number of prescribing omissions (OR=0.90; 95% CI=0.41 to 1.96). The per-protocol analysis showed no statistically significant group difference either and there were no group differences in the secondary outcomes. In 59% of participants at least one prescribing recommendation to stop or start a medication was implemented. It is of note that not all prescribing recommendations generated by STRIPA were accepted by GPs and discussed with patients. The OPTICA trial was approved by the Cantonal Ethics Committee of the Canton of Bern (*BASEC-ID: 2018–00914*). All participants or their legal representatives provided written informed consent.

Study design and Sample Definition

This is a longitudinal, post-hoc sub-study of data collected during the OPTICA trial. Data from the trial baseline, the 6-month follow-up, and the 12-month follow-up were used for the present analyses. This manuscript adheres to the Strengthening the Reporting of Observational Studies in Epidemiology (STROBE) reporting guideline for observational studies.[27] All 323 participants of the OPTICA trial were older adults (≥65 years of age), with multimorbidity (≥3 chronic conditions) and polypharmacy (≥5 medications). We limited the present analyses to the participants for whom the patient version of the 'revised Patient Attitudes Towards Deprescribing' (rPATD) was used (N = 298).[16]

Assessment of patients' willingness to have medications deprescribed

Patients' attitudes towards having medications deprescribed hypothetically was measured using the rPATD at baseline. The rPATD contains 22 questions with "Strongly disagree (1)" and "Strongly agree (5)" as the scale anchors.[16] For the main analyses, we used the global question from the rPATD "If my doctor said it was possible, I would be willing to stop one or more of my regular medicines" as the independent variable, which measures patients' willingness to accept deprescribing proposed by a medical doctor. In addition, we used two questions from the rPATD "appropriateness" factor ("I would like to try stopping one of my medicines to see how I feel without

For peer review only - http://bmjopen.bmj.com/site/about/guidelines.xhtml

it" and "I would like my doctor to reduce the dose of one or more of my medicines"), which aim to measure patients' desires to try to stop or reduce medicines (Box 1). Further, we used the rPATD "concerns about stopping" factor score (ranging from 1 to 5) calculated based on five rPATD questions as independent variables. Several questions from the rPATD were used as independent variables given the ceiling effect of the global rPATD willingness to deprescribe question and the fact that there is more variation in the responses to the other two rPATD questions and the concerns about stopping score.

Box 1: Attitudes towards deprescribing: rPATD questions

**Global question:**<br>*"If my doctor said it was possible, I would be willing to stop one or more of my regular medicines"*
**Appropriateness questions:**<br>*"I would like to try stopping one of my medicines to see how I feel without it"*<br>*"I would like my doctor to reduce the dose of one or more of my medicines"*
**Concerns about stopping questions:**<br>*"I have had a bad experience when stopping a medicine before"*<br>*"I would be reluctant to stop a medicine that I had been taking for a long time"*<br>*"If one of my medicines was stopped I would be worried about missing out on future benefits"*<br>*"I get stressed whenever changes are made to my medicines"*<br>*"If my doctor recommended stopping a medicine I would feel that he/she was giving up on me"*

Assessment of medication-related outcomes over time

Medication-related outcomes over time were assessed using data collected at baseline and throughout the follow-up period of the OPTICA trial. Details on how the three medication-related outcomes were assessed – change in the number of medications, medication appropriateness, and the implementation of prescribing recommendations – can be found in **Box 2**.

For peer review only - http://bmjopen.bmj.com/site/about/guidelines.xhtml

Box 2: Assessment of medication-related outcomes

	Outcome	Description	Measurement
**Aim 1**	Number of long-term medications	Integer number of medications used for ≥90 days	Change in the number of long-term medications (≥90 days, "as needed" medications were excluded) between baseline and the 12-month follow-up.
**Aim 2**	Medication appropriateness	The Medication Appropriateness Index (MAI) [28] is an implicit tool for assessing the appropriateness of medication prescribing. The 10-item version of the MAI was used as one of the co-primary outcomes of the OPTICA trial, however, the cost effectiveness item was excluded for feasibility reasons. Using data on medications, diagnoses, and lab values the assessors rated the nine remaining criteria of the MAI for each medication used for ≥90 days using a three-point scale ranging from A=appropriate, B=marginally appropriate, to C=inappropriate.	Change in the average medication appropriateness between baseline and the 12-month follow-up.<br>We first calculated the average MAI for the baseline and the 12-month follow-up by dividing the total MAI score of the respective timepoint by the number of long-term medications at this timepoint. Then we calculated the change in the average MAI between baseline and the 12-month follow-up.
**Aim 3**	Implementation of prescribing recommendations to stop medications	Recommendation implemented yes vs. no	Binary variable describing whether any deprescribing recommendation to stop a medication generated by the electronic decision support system tested in the OPTICA trial had been implemented or not at the patient level. Only data from the OPTICA intervention group was used.

For peer review only - http://bmjopen.bmj.com/site/about/guidelines.xhtml

Co-variates

The following variables were used to adjust the analyses: gender, age, educational status, number of chronic conditions, living situation, capable of leaving the house (yes/no), patients' satisfaction with medications, and number of GP visits in the 6 months prior to the study enrolment. The included variables were based on the literature of the factors associated with number of medications/polypharmacy and the factors associated with potentially inappropriate medication use/medication appropriateness considering the data available from the OPTICA trial.[29-35]

Statistical analysis

First, we described the demographics and main clinical characteristics of the study participants. Second, we descriptively analysed three questions from the rPATD and the concerns about stopping score to describe patients' willingness to have medications deprescribed at baseline. Third, we performed a set of multilevel regression analyses. For Aims 1 and 2, we used multilevel linear regression models to investigate the association between patients' willingness to have medications deprescribed and the outcomes. In subgroup analyses, we restricted the analyses to the OPTICA intervention group. For Aim 3, we used a multilevel logistic regression model to investigate the association between patients' willingness to have medications deprescribed and the binary outcome variable. For Aim 3, we used data from the OPTICA intervention group only. All analyses were adjusted for the clustering effect at the GP level and the measurable co-variates listed in the section above plus the group allocation during the trial (except for the analyses for Aim 3, which were based on data from the intervention group only). Analyses were limited to the observed data, and we did not use any multiple imputation methods. All analyses were performed with STATA 15.1 (StataCorp, College Station, TX, USA).

Patient and public involvement

No patients were involved in setting the research question or the outcome measures of the OPTICA trial. General practitioners and patients aged ≥65 years with multimorbidity and polypharmacy were represented in the Safety and Data Monitoring Board. General practitioners and patients who participated in the trial received newsletters throughout the trial.

For peer review only - http://bmjopen.bmj.com/site/about/guidelines.xhtml

Results

Baseline characteristics

Table 1 describes the baseline characteristics of study participants**.** Out of the 298 participants for whom information on their willingness to have medications deprescribed was assessed at baseline (92% of all trial participants), 45% were women and the median age was 78 years. 75% (224/298) of the participants had equal or higher than median willingness to have medications deprescribed.

For peer review only

For peer review only - http://bmjopen.bmj.com/site/about/guidelines.xhtml

Table 1. Baseline characteristics of study participants by willingness to deprescribe (n = 298)

	All patients in the sample *(n=298)*[2]	Patients with lower than median willingness to deprescribe *(n=74)*[1]	Patients with equal or higher than median willingness to deprescribe *(n=224)*[1]
Age (in years)	78 (74-83)	79 (74-83)	78 (74-83)
Female	133 (45)	39 (53)	94 (42)
Patient education			
Mandatory schooling or less	113 (38)	25 (34)	86 (39)
Diploma at secondary school level	139 (47)	33 (45)	106 (47)
Higher education diploma	45 (15)	16 (22)	29 (13)
Number of chronic conditions	7 (5-10)	7 (5-9)	7 (5-11)
Living situation			
In apartment/ house without any external help	227 (76)	62 (84)	165 (74)
In apartment/ house with some external help	61 (20)	9 (12)	52 (23)
In a nursing home	10 (3)	3 (4)	7 (3)
Patient is unable to leave the house (as compared to not housebound)	7 (2)	2 (3)	5 (2)
Equal or higher than median satisfaction with current medication use (as compared to lower than medication willingness to deprescribe)	215 (72)	59 (80)	156 (70)
Number of GP consultations during the 6-month follow-up period prior to the enrolment into the study trial	8 (5-14)	9 (6-13)	8 (5-15)
Average Medication Appropriateness Index at baseline	1.7 (0.2-5)	1.8 (0.2-6)	1.7 (0.2-4.7)
Number of long-term medications	8 (5-11)	8 (5-10)	8 (5-11)

For continuous variables the median and the interquartile range (IQR) are presented. For categorial variables frequencies and percentages are presented. | [1] Patients' willingness to have medications deprescribed was measured using the rPATD global question "If my doctor said it was possible, I would be willing to stop one or more of my regular medicines". The median willingness to have medications deprescribed corresponded to "strongly agree" with the rPATD global question. | [2] Among the 298 patients, 146 patients were then randomized to the control group and 152 patients to the intervention group. Among patients with lower than median willingness to deprescribe 36 were randomized to the control group and 38 to the intervention group. Among patients with equal or higher than median willingness to deprescribe 110 were assigned to the control group and 114 were randomized to the intervention group. | Missingness: Gender, and age had 0% missing values. Patient education, living situation, housebound yes/no, patient satisfaction with medications, and the number of chronic medications had less than 3% missing information. The number of chronic conditions and the average Medication Appropriateness Index at baseline had less than 7% missing.

For peer review only - http://bmjopen.bmj.com/site/about/guidelines.xhtml

Proxy measures for patients' willingness to have medications deprescribed

Table 2 shows the descriptive results of the different measures used to assess patients' willingness to have medications deprescribed. More than 85% of participants strongly agreed or agreed with the rPATD global question and only 9% of participants disagreed with this statement, whereas there was slightly more variation in responses to the other two rPATD questions. Approximately 60% of participants reported they would like to try stopping one of their medications to see how they would feel without it, whereas 32% disagreed or strongly disagreed with this statement.

Table 2. Patients' attitudes towards having medications deprescribed at baseline[1] (n=298)

rPATD global question: "If my doctor said it was possible, I would be willing to stop one or more of my regular medicines" frequency (percent)				
Strongly agree	Agree	Unsure	Disagree	Strongly disagree
224 (75)	38 (13)	9 (3)	14 (5)	13 (4)
Alternative measurements of patients' willingness to have medications deprescribed based on the rPATD				
Concerns about stopping score		Mean (SD)	Median (IQR)	
		1.8 (0.8)	1.6 (1-2.4)	
"I would like to try stopping one of my medicines to see how I feel without it"				
Strongly agree	Agree	Unsure	Disagree	Strongly disagree
120 (40)	65 (22)	19 (6)	59 (20)	35 (12)
"I would like my doctor to reduce the dose of one or more of my medicines"				
Strongly agree	Agree	Unsure	Disagree	Strongly disagree
153 (51)	64 (22)	24 (8)	29 (10)	28 (9)

[1] As measured by the 'revised Patients' Attitudes Towards Deprescribing (rPATD) questionnaire. Source: Reeve, E., al. Development and Validation of the Revised Patients' Attitudes Towards Deprescribing (rPATD) Questionnaire: Versions for Older Adults and Caregivers. Drugs Aging 33, 913–928 (2016). | Missingness: There was 0% missingness in rPATD questions and the concerns of stopping score.

Number of medications over time

Table 3 shows the associations between the different measures assessing patients' willingness to have medications deprescribed and the change in number of medications throughout the 12-month follow-up period. At the 12-month follow-up, the mean change in the number of medications was -0.2 (standard deviation=4.2). The only statistically significant association was between the concerns about stopping score and the change in the number of medications (coefficient: 0.65, 95% CI: 0.08 to 1.22). A higher score indicates being more concerned about stopping. Meaning, per 1-unit increase in the concerns about stopping score the change in the number of medications between baseline and the 12-month follow-up increased by 0.65. In the sub-group analyses restricted to participants in the intervention group the results were identical (**eTable 1**).

For peer review only - http://bmjopen.bmj.com/site/about/guidelines.xhtml

Table 3. Multivariate associations between the change in the number of medications throughout the 12-month follow-up period and patients' willingness to deprescribe (n=253)

Name of the variable	*Coefficient*	*p-value*	*95% confidence interval*
rPATD global question: "If my doctor said it was possible, I would be willing to stop one or more of my regular medicines" (reference: strongly agree)			
agree	-0.96	0.169	-2.33 to 0.41
unsure	0.61	0.963	-2.52 to 2.64
disagree	0.58	0.598	-1.56 to 2.71
strongly disagree	0.26	0.806	-1.81 to 2.33
Alternative measurements of patients' willingness to have medications deprescribed based on the rPATD			
Concerns about stopping score (per 1-unit increase)[1]	0.65	**0.026**	0.08 to 1.22
'I would like to try stopping one of my medicines to see how I feel without it' (reference: strongly agree)			
agree	-0.12	0.830	-1.33 to 1.07
unsure	0.62	0.509	-1.24 to 2.51
disagree	0.47	0.448	-0.74 to 1.68
strongly disagree	-0.21	0.774	-1.68 to 1.25
'I would like my doctor to reduce the dose of one or more of my medicines' (reference: strongly agree)			
agree	1.13	0.070	-0.09 to 2.36
unsure	-0.97	0.251	-2.64 to 0.69
disagree	0.79	0.306	-0.72 to 2.31
strongly disagree	0.71	0.359	-0.81 to 2.24

Multilevel linear regression models adjusted for patient age, education status, gender, number of chronic conditions, living situation, whether the patient is housebound or not, patient satisfaction with medications, the number of GP consultations in the 6-months prior to the study inclusion, and the group allocation during the trial. | [1]As calculated based on Reeve, E., al. Development and Validation of the Revised Patients' Attitudes Towards Deprescribing (rPATD) Questionnaire: Versions for Older Adults and Caregivers. Drugs Aging 33, 913–928 (2016). | Missingness: The change in the number of chronic medications over the 12-month follow-up period had 8% missing values.

Medication appropriateness over time

The associations between patients' willingness to have medications deprescribed and the change in medication appropriateness throughout the 12-month follow-up period is shown in **Table 4**. At the 12-month follow-up, the mean change in the average Medication Appropriateness Index was -0.75 (Standard deviation=2.5). We did not find evidence for the statistically significant associations. In the subgroup analyses restricted to the OPTICA intervention group, we found statistically significant associations between patients' being undecided or (strongly) agreeing with the statement "I would like my doctor to reduce the dose of one or more of my medicines" and an improvement in medication appropriateness between baseline and the 12-month follow-up period (results not presented).

For peer review only - http://bmjopen.bmj.com/site/about/guidelines.xhtml

Table 4. Multivariate associations between the change in the medication appropriateness[1] throughout the 12-month follow-up period and patients' willingness to deprescribe[2] (n=242)

Name of the variable	*Coefficient*	*p-value*	*95% confidence interval*
rPATD global question: "If my doctor said it was possible, I would be willing to stop one or more of my regular medicines" (reference: strongly agree)			
agree	0.35	0.426	-0.51 to 1.21
unsure	0.92	0.293	-0.79 to 2.63
disagree	-1.01	0.145	-2.36 to 0.35
strongly disagree	-0.80	0.221	-2.08 to 0.48
Alternative measurements of patients' willingness to have medications deprescribed based on the rPATD			
Concerns about stopping score (per 1-unit increase)[1]			
	-0.29	0.120	-0.65 to 0.08
'I would like to try stopping one of my medicines to see how I feel without it' (reference: strongly agree)			
agree	-0.45	0.253	-1.21 to 0.32
unsure	-0.66	0.281	-1.87 to 0.54
disagree	-0.45	0.246	-1.22 to 0.31
strongly disagree	-0.57	0.233	-1.51 to 0.37
'I would like my doctor to reduce the dose of one or more of my medicines' (reference: strongly agree)			
agree	-0.44	0.253	-1.20 to 0.32
unsure	-0.59	0.282	-1.67 to 0.49
disagree	-0.02	0.968	-0.95 to 0.99
strongly disagree	0.13	0.795	-0.85 to 1.11

Multilevel linear regression models adjusted for patient age, education status, gender, number of chronic conditions, living situation, whether the patient is housebound or not, patient satisfaction with medications, the number of GP consultations in the 6-months prior to the study inclusion, and the group allocation during the trial. | [1]As assessed using the Medication Appropriateness Index: Samsa GP, Hanlon JT, Schmader KE, Weinberger M, Clipp EC, Uttech KM, Lewis IK, Landsman PB, Cohen HJ. A summated score for the medication appropriateness index: development and assessment of clinimetric properties including content validity. J Clin Epidemiol. 1994 Aug;47(8):891-6. | [2]As calculated based on Reeve, E., al. Development and Validation of the Revised Patients' Attitudes Towards Deprescribing (rPATD) Questionnaire: Versions for Older Adults and Caregivers. Drugs Aging 33, 913–928 (2016). | Missingness: The change in the Medication Appropriateness Index over the 12-month follow-up period had 13% values.

Implementation of prescribing recommendations

Table 5 shows the association between patients' willingness to have medications deprescribed and the implementation of prescribing recommendations that were generated as part of the OPTICA medication review intervention (n=31). On average, 1 prescribing recommendation to stop or start a medication were reported to be implemented per patient (reported elsewhere [36]) and 59% of patients in the intervention group had 1 or more prescribing recommendation implemented. We did not find any evidence for a statistically significant association between patients' willingness to have medications deprescribed and the implementation of deprescribing recommendations.

For peer review only - http://bmjopen.bmj.com/site/about/guidelines.xhtml

Table 5. Multivariate associations between the implementation of recommendations to stop medications and patients' willingness to deprescribe[1] (n=31)

Name of the variable	*Odds ratio*	*p-value*	*95% confidence interval*
rPATD global question: "If my doctor said it was possible, I would be willing to stop one or more of my regular medicines" (reference: equal or higher than median agreement)[2]			
Below median agreement	4.90	0.244	0.34 to 71.3
Alternative measurements of patients' willingness to have medications deprescribed based on the rPATD			
Concerns about stopping score (per 1-unit increase)			
	1.13	0.812	0.41 to 3.13
'I would like to try stopping one of my medicines to see how I feel without it' (reference: equal or higher than median agreement)[2]			
Below median agreement	2.53	0.305	0.43 to 14.89
'I would like my doctor to reduce the dose of one or more of my medicines' (reference: equal or higher than median agreement) [2]			
Below median agreement	7.82	0.086	0.75 to 82.2

[1] Multilevel logistic regression models adjusted for patient age, and gender. | [2] Due to the low number of observations for which the implementation of recommendations was reported, the rPATD question was dichotomized.
The analyses presented in this table used data from the OPTICA intervention group only. Despite several reminders, only a couple of general practitioners from the OPTCIA intervention group reported this information.

Discussion

In this sub-study of a cluster randomised clinical trial, we examined the association between older adults' hypothetical willingness to have one or more medications deprescribed and change in a participant's medications, appropriateness of their medications, and actual implementation of prescribing recommendations. Overall, we found that these medication-related outcomes measured over one year were not associated with the rPATD deprescribing questions measured in this study. To consider reasons why no association was found, firstly we discuss the rPATD questions in more detail and their ability to measure self-reported attitudes towards deprescribing. Secondly, consideration is given to our deprescribing intervention and how medication-related outcomes were measured in this study.

In our study, 88% of participants agreed or strongly agreed with the rPATD global question: "If my doctor said it was possible, I would be willing to have one or more of my medications deprescribed". However, this high agreement was not associated with changes in medication-related outcomes over time. Other deprescribing intervention trials using the (r)PATD global question [37-39] also found high agreement with hypothetical willingness to deprescribe (86-95%) with no effect on deprescribing or medication-related outcomes. The majority of studies using the rPATD global deprescribing question report greater than 80% agreement [8] therefore, it may be difficult to find an association given the ceiling effect of this question.

For peer review only - http://bmjopen.bmj.com/site/about/guidelines.xhtml

A recent cluster randomised controlled trial conducted in Ireland with older adults taking ≥15 medications found that a higher willingness to deprescribe measured by the rPATD was not only associated with a higher rate of deprescribing but also initiating medicines.[17] The authors note that the rPATD global question may identify participants who are agreeable to any medication-changes if they are suggested by their doctor. Supporting this, there is variation between the global question and another rPATD deprescribing question which does not refer to the doctor.[8] In our study, agreement was much higher for the rPATD global deprescribing question with 88% of participants willing to deprescribe if their doctor said it was possible, however 62% wanted to try stopping one of their medications to see how they would feel without it. Other studies using the rPATD have shown substantial differences (30-73% gap) between these questions with the global question responses always higher [8, 40-44] suggesting the influence of the doctor should not be underestimated. Similarly, a content analysis including over 2,500 participants from Australia, the UK and the US found that approximately one-half of older adults who agreed with deprescribing in a hypothetical scenario felt that the doctor's recommendation was an important consideration.[45]

There is a complex interplay of factors, such as clinical decision-making and patients' attitudes, that are behind acceptance (or not) of deprescribing. It is possible that the lack of association between the rPATD and medication-related changes in our study was due to the inconclusive effectiveness of the OPTICA deprescribing intervention, which is similar to other deprescribing interventional studies. While it is useful to quantify attitudes towards deprescribing to get a sense of older adults' general thoughts about their medications, it may be unfair to expect self-reported attitudes to equate to actual medication changes. Of note, an exploratory deprescribing controlled trial conducted in Australia measured the original PATD (10 questions) at baseline and again at follow-up.[46] Although the PATD baseline scores did not predict deprescribing outcomes, statistically significant changes were shown in 3 questions which signalled a shift in patients' beliefs about medicines.

Deprescribing in clinical practice and interventional studies may not occur for many reasons, such as if the general practitioner chooses not to initiate it. From the main OPTICA trial, the most common reasons for not implementing prescribing recommendations were that general practitioners thought that patients' current medications were beneficial and that the recommended change was not

For peer review only - http://bmjopen.bmj.com/site/about/guidelines.xhtml

suitable. Therefore, there is a need to capture the reasons behind patients or doctors resisting deprescribing in interventional and survey studies, as this is a gap in deprescribing research.

Further exploration is needed into the link between attitudes towards medicines and actual medication changes, possibly through process evaluations of deprescribing trials. Attitudinal measures of deprescribing may benefit from greater sensitivity to reluctance towards deprescribing, less vulnerability to the doctor's influence, and capturing attitudes towards specific medications.[44, 47] Ultimately, it would be useful for a tool to identify patients at different degrees of willingness to deprescribe so that deprescribing interventions can be tailored to their needs and preferences.

Strengths and Limitations

The present analyses were strengthened by the longitudinal design, which allows for a clear temporal distinction between patients' willingness to have medications deprescribed assessed at baseline and the medication-related outcomes over time in addition to offer the intervention to optimize medication randomly. We would like to emphasize the following limitations of these present analyses. Firstly, patients agreeing to participate in the OPTICA trial could have had a higher willingness to have one or more of their medications deprescribed. Due to challenges with how data from the electronic health records of participating patients were recorded, there was some missing data on medication, which is why some participants were excluded from the analyses. Also, to determine the medication-related outcomes for aim 1 and 2 we used prescribing data from electronic health records, which does not necessarily correspond to what medications were actually used by patients. Finally, despite several reminders, only a small proportion of GPs from the intervention group reported which prescribing recommendations were implemented together with patients. This explains the smaller sample size for our third aim. Due to the small sample size used to analyse Aim 3, the confidence intervals were wide and imprecise.

Conclusions

Our findings indicate that there is no association between patients' willingness to have medications deprescribed and medication-related outcomes over time. It is important to capture a range of participant attitudes that are both for and against deprescribing, as well as to consider the

For peer review only - http://bmjopen.bmj.com/site/about/guidelines.xhtml

For peer review only

relationship between self-report surveys and actual deprescribing. The results highlight the need for further research to better understand the factors that contribute to successful deprescribing in primary care settings.

For peer review only

For peer review only - http://bmjopen.bmj.com/site/about/guidelines.xhtml

Declarations

Data sharing statement

We will make the data for this study available to other researchers upon request after publication. The data will be made available for scientific research purposes, after the proposed analysis plan has been approved. Data and documentation will be made available through a secure file exchange platform after approval of the proposal. In addition, a data transfer agreement must be signed (which defines obligations that the data requester must adhere to with regard to privacy and data handling). Deidentified participant data limited to the data used for the proposed project will be made available, along with a data dictionary and annotated case report forms. For data access, please contact the corresponding author.

Ethical approval

The study protocol of the OPTICA trial and other documentation was approved by the competent ethics committee of the canton of Bern (KEK), Switzerland, and the Swiss regulatory authority (Swissmedic) (BASEC ID: 2018–00914). The KEK and Swissmedic received annually safety reports and were informed about the end of the study. All participants gave their written informed consent. The OPTICA trial was performed in accordance with relevant regulations and guidelines.

Funding

The OPTICA trial was supported by the Swiss National Science Foundation, within the framework of the National Research Programme 74 "Smarter Health Care" (NRP74) under contract number 407440_167465 (to Prof Streit, Prof Rodondi, and Prof Schwenkglenks). Dr Jungo was funded by a Postdoc.Mobility Fellowship from the Swiss National Science Foundation (P500PM_206728). Dr Weir was funded by a Swiss Government Excellence Scholarship (n/a), a Swiss National Science Foundation Scientific Exchanges grant (IZSEZ0_213475) and a NHMRC Emerging Leader Research Fellowship (2017295).

For peer review only - http://bmjopen.bmj.com/site/about/guidelines.xhtml

Role of the Funder

The funders had no role in the design and conduct of the study; collection, management, analysis, and interpretation of the data; preparation, review, or approval of the manuscript; and decision to submit the manuscript for publication.

Conflict of interest disclosures

The authors do not have any conflicts of interest to disclose.

Acknowledgements

We would like to thank the GPs who participated in the OPTICA trial for their great contributions without which it would have been impossible to conduct this study. We also thank the participants for consenting to participate in our study. Dr Jungo is a member of the Junior Investigator Intensive Program of the US Deprescribing Research Network, which is funded by the National Institute on Aging.

Author contributions

All authors contributed to the concept and study design. All authors contributed to the acquisition, analysis, or interpretation of data. KTJ and KRW wrote the first draft of the manuscript. All other authors provided feedback and approved the final version of the manuscript. KTJ and SS provided administrative and technical support. KTJ did the statistical analyses. SS obtained funding for the work and supervised the study. KTJ and KRW had full access to all the data in the study and are the guarantors.

For peer review only

For peer review only - http://bmjopen.bmj.com/site/about/guidelines.xhtml

References

1. Maher RL, Hanlon J, Hajjar ER. Clinical consequences of polypharmacy in elderly. Expert Opinion on Drug Safety. 2014;13(1):57-65.
2. Fried TR, O'Leary J, Towle V, Goldstein MK, Trentalange M, Martin DK. Health outcomes associated with polypharmacy in community-dwelling older adults: a systematic review. J Am Geriatr Soc. 2014;62(12):2261-72.
3. Kantor ED, Rehm CD, Haas JS, Chan AT, Giovannucci EL. Trends in Prescription Drug Use Among Adults in the United States From 1999-2012. Jama. 2015;314(17):1818-31.
4. Jungo KT, Streit S, Lauffenburger JC. Utilization and Spending on Potentially Inappropriate Medications by US Older Adults with Multiple Chronic Conditions using Multiple Medications. Archives of Gerontology and Geriatrics. 2021;93:104326.
5. American Geriatrics Society 2019 Updated AGS Beers Criteria® for Potentially Inappropriate Medication Use in Older Adults. J Am Geriatr Soc. 2019;67(4):674-94.
6. Scott IA, Hilmer SN, Reeve E, Potter K, Le Couteur D, Rigby D, et al. Reducing Inappropriate Polypharmacy: The Process of DeprescribingReducing Inappropriate PolypharmacyReducing Inappropriate Polypharmacy. JAMA Internal Medicine. 2015;175(5):827-34.
7. Weir K, Nickel B, Naganathan V, Bonner C, McCaffery K, Carter SM, et al. Decision-Making Preferences and Deprescribing: Perspectives of Older Adults and Companions About Their Medicines. J Gerontol B Psychol Sci Soc Sci. 2018;73(7):e98-e107.
8. Weir KR, Ailabouni NJ, Schneider CR, Hilmer SN, Reeve E. Consumer attitudes towards deprescribing: a systematic review and meta-analysis. The journals of gerontology Series A, Biological sciences and medical sciences. 2021.
9. Weir KR, Naganathan V, Carter SM, Tam CWM, McCaffery K, Bonner C, et al. The role of older patients' goals in GP decision-making about medicines: a qualitative study. BMC Family Practice. 2021;22(1):13.
10. Ailabouni NJ, Nishtala PS, Mangin D, Tordoff JM. General practitioners' insight into deprescribing for the multimorbid older individual: a qualitative study. International Journal of Clinical Practice. 2016;70(3):261-76.
11. Zechmann S, Senn O, Valeri F, Essig S, Merlo C, Rosemann T, et al. Effect of a patient-centred deprescribing procedure in older multimorbid patients in Swiss primary care - A cluster-randomised clinical trial. BMC geriatrics. 2020;20(1):471.
12. Kutner JS, Blatchford PJ, Taylor DH, Ritchie CS, Bull JH, Fairclough DL, et al. Safety and benefit of discontinuing statin therapy in the setting of advanced, life-limiting illness: a randomized clinical trial. JAMA internal medicine. 2015;175(5):691-700.
13. McCarthy C, Flood M, Clyne B, Smith SM, Boland F, Wallace E, et al. Association between patient attitudes towards deprescribing and subsequent prescription changes. Basic & Clinical Pharmacology & Toxicology. 2023.
14. Vasilevskis EE, Shah AS, Hollingsworth EK, Shotwell MS, Kripalani S, Mixon AS, et al. Deprescribing Medications Among Older Adults From End of Hospitalization Through Postacute Care: A Shed-MEDS Randomized Clinical Trial. JAMA Internal Medicine. 2023;183(3):223-31.
15. Linsky AM, Stolzmann K, Meterko M. The Patient Perceptions of Deprescribing (PPoD) Survey: Short-Form Development. Drugs Aging. 2020;37(12):909-16.
16. Reeve E, Low LF, Shakib S, Hilmer SN. Development and Validation of the Revised Patients' Attitudes Towards Deprescribing (rPATD) Questionnaire: Versions for Older Adults and Caregivers. Drugs Aging. 2016;33(12):913-28.
17. McCarthy C, Flood M, Clyne B, Smith SM, Boland F, Wallace E, et al. Association between patient attitudes towards deprescribing and subsequent prescription changes (preprint). 2023.
18. Jungo KT, Rozsnyai Z, Mantelli S, Floriani C, Löwe AL, Lindemann F, et al. 'Optimising PharmacoTherapy In the multimorbid elderly in primary CAre' (OPTICA) to improve medication appropriateness: study protocol of a cluster randomised controlled trial. BMJ open. 2019;9(9):e031080.
19. Jungo KT, Meier R, Valeri F, Schwab N, Schneider C, Reeve E, et al. Baseline characteristics and comparability of older multimorbid patients with polypharmacy and general practitioners participating in a randomized controlled primary care trial. BMC Family Practice. 2021;22(1):123.

For peer review only - http://bmjopen.bmj.com/site/about/guidelines.xhtml

20. Jungo KT, Ansorg A-K, Floriani C, Rozsnyai Z, Schwab N, Meier R, et al. Optimising prescribing in older adults with multimorbidity and polypharmacy in primary care (OPTICA): cluster randomised clinical trial. bmj. 2023;381.
21. Drenth-van Maanen AC, Leendertse AJ, Jansen PAF, Knol W, Keijsers C, Meulendijk MC, et al. The Systematic Tool to Reduce Inappropriate Prescribing (STRIP): Combining implicit and explicit prescribing tools to improve appropriate prescribing. J Eval Clin Pract. 2018;24(2):317-22.
22. Meulendijk MC, Spruit MR, Drenth-van Maanen AC, Numans ME, Brinkkemper S, Jansen PAF, et al. Computerized Decision Support Improves Medication Review Effectiveness: An Experiment Evaluating the STRIP Assistant's Usability. Drugs & Aging. 2015;32(6):495-503.
23. Meulendijk MC, Spruit MR, Willeboordse F, Numans ME, Brinkkemper S, Knol W, et al. Efficiency of Clinical Decision Support Systems Improves with Experience. Journal of medical systems. 2016;40(4):76.
24. Hanlon JT, Schmader KE, Ruby CM, Weinberger M. Suboptimal prescribing in older inpatients and outpatients. J Am Geriatr Soc. 2001;49(2):200-9.
25. Hanlon JT, Schmader KE, Samsa GP, Weinberger M, Uttech KM, Lewis IK, et al. A method for assessing drug therapy appropriateness. Journal of clinical epidemiology. 1992;45(10):1045-51.
26. Samsa GP, Hanlon JT, Schmader KE, Weinberger M, Clipp EC, Uttech KM, et al. A summated score for the medication appropriateness index: development and assessment of clinimetric properties including content validity. Journal of clinical epidemiology. 1994;47(8):891-6.
27. von Elm E, Altman DG, Egger M, Pocock SJ, Gøtzsche PC, Vandenbroucke JP. The Strengthening the Reporting of Observational Studies in Epidemiology (STROBE) statement: guidelines for reporting observational studies. Journal of clinical epidemiology. 2008;61(4):344-9.
28. Samsa GP, Hanlon JT, Schmader KE, Weinberger M, Clipp EC, Uttech KM, et al. A summated score for the medication appropriateness index: development and assessment of clinimetric properties including content validity. Journal of clinical epidemiology. 1994;47(8):891-6.
29. Cho HJ, Chae J, Yoon SH, Kim DS. Factors related to polypharmacy and hyper-polypharmacy for the elderly: A nationwide cohort study using National Health Insurance data in South Korea. Clinical and Translational Science. 2023;16(2):193-205.
30. Delara M, Murray L, Jafari B, Bahji A, Goodarzi Z, Kirkham J, et al. Prevalence and factors associated with polypharmacy: a systematic review and meta-analysis. BMC geriatrics. 2022;22(1):601.
31. Nothelle SK, Sharma R, Oakes A, Jackson M, Segal JB. Factors associated with potentially inappropriate medication use in community-dwelling older adults in the United States: a systematic review. International Journal of Pharmacy Practice. 2019;27(5):408-23.
32. Slater N, White S, Venables R, Frisher M. Factors associated with polypharmacy in primary care: a cross-sectional analysis of data from The English Longitudinal Study of Ageing (ELSA). BMJ open. 2018;8(3):e020270.
33. Tian F, Chen Z, Zeng Y, Feng Q, Chen X. Prevalence of Use of Potentially Inappropriate Medications Among Older Adults Worldwide: A Systematic Review and Meta-Analysis. JAMA Network Open. 2023;6(8):e2326910-e.
34. Xu Z, Liang X, Zhu Y, Lu Y, Ye Y, Fang L, et al. Factors associated with potentially inappropriate prescriptions and barriers to medicines optimisation among older adults in primary care settings: a systematic review. Family Medicine and Community Health. 2021;9(4).
35. Ye L, Yang-Huang J, Franse CB, Rukavina T, Vasiljev V, Mattace-Raso F, et al. Factors associated with polypharmacy and the high risk of medication-related problems among older community-dwelling adults in European countries: a longitudinal study. BMC geriatrics. 2022;22(1):1-13.
36. Jungo KT, Ansorg A-K, Floriani C, Rozsnyai Z, Schwab N, Meier R, et al. Optimizing Prescribing in Older Adults with Multimorbidity and Polypharmacy in Primary Care: A Cluster Randomized Clinical Trial (OPTICA Trial). medRxiv. 2022:2022.10.31.22281164.
37. Kouladjian O'Donnell L, Gnjidic D, Sawan M, Reeve E, Kelly PJ, Chen TF, et al. Impact of the Goal-directed Medication Review Electronic Decision Support System on Drug Burden Index: A cluster-randomised clinical trial in primary care. Br J Clin Pharmacol. 2020.
38. Anderson K, Freeman C, Foster M, Scott I. GP-Led Deprescribing in Community-Living Older Australians: An Exploratory Controlled Trial. J Am Geriatr Soc. 2020;68(2):403-10.
39. Turner JP, Martin P, Zhang YZ, Tannenbaum C. Patients beliefs and attitudes towards deprescribing: Can deprescribing success be predicted? Research in social & administrative pharmacy : RSAP. 2020;16(4):599-604.

For peer review only - http://bmjopen.bmj.com/site/about/guidelines.xhtml

40. Rozsnyai Z, Jungo KT, Reeve E, Poortvliet RKE, Rodondi N, Gussekloo J, et al. What do older adults with multimorbidity and polypharmacy think about deprescribing? The LESS study - a primary care-based survey. BMC geriatrics. 2020;20(1):435.
41. Navid P, Nguyen L, Jaber D, Zarzuela K, Musse M, Lu Wang M, et al. Attitudes toward deprescribing among adults with heart failure with preserved ejection fraction. J Am Geriatr Soc. 2021;69(7):1948-55.
42. Crutzen S, Abou J, Smits SE, Baas G, Hugtenburg JG, Heringa M, et al. Older people's attitudes towards deprescribing cardiometabolic medication. BMC geriatrics. 2021;21(1):366.
43. Lukacena KM, Keck JW, Freeman PR, Harrington NG, Huffmyer MJ, Moga DC. Patients' attitudes toward deprescribing and their experiences communicating with clinicians and pharmacists. Therapeutic advances in drug safety. 2022;13:20420986221116465.
44. Bužančić I, Dragović P, Pejaković TI, Markulin L, Ortner-Hadžiabdić M. Exploring Patients' Attitudes Toward Deprescribing and Their Perception of Pharmacist Involvement in a European Country: A Cross-Sectional Study. Patient preference and adherence. 2021;15:2197-208.
45. Vordenberg SE, Rana R, Shang J, Choi J, Scherer AM, Weir KR. Reasons why older adults in three countries agreed with a deprescribing recommendation in a hypothetical vignette. Basic & clinical pharmacology & toxicology. 2023.
46. Anderson K, Freeman C, Foster M, Scott I. GP-led deprescribing in community-living older Australians: an exploratory controlled trial. Journal of the American Geriatrics Society. 2020;68(2):403-10.
47. Lüthold RV, Jungo KT, Weir KR, Geier A-K, Scholtes B, Kurpas D, et al. Understanding older patients' willingness to have medications deprescribed in primary care: a protocol for a cross-sectional survey study in nine European countries. BMC geriatrics. 2022;22(1):920.

For peer review only

For peer review only - http://bmjopen.bmj.com/site/about/guidelines.xhtml

STROBE Statement—checklist of items that should be included in reports of observational studies

	Item No	Recommendation
**Title and abstract**	1	(*a*) Indicate the study's design with a commonly used term in the title or the abstract ✓ Page 2
		(*b*) Provide in the abstract an informative and balanced summary of what was done and what was found ✓ Page 2
**Introduction**		
Background/rationale	2	Explain the scientific background and rationale for the investigation being reported ✓ Page 4
Objectives	3	State specific objectives, including any prespecified hypotheses
**Methods**		
Study design	4	Present key elements of study design early in the paper ✓ Page 5
Setting	5	Describe the setting, locations, and relevant dates, including periods of recruitment, exposure, follow-up, and data collection ✓ Page 5-6
Participants	6	(*a*) *Cohort study*—Give the eligibility criteria, and the sources and methods of selection of participants. Describe methods of follow-up *Case-control study*—Give the eligibility criteria, and the sources and methods of case ascertainment and control selection. Give the rationale for the choice of cases and controls *Cross-sectional study*—Give the eligibility criteria, and the sources and methods of selection of participants
		(*b*) *Cohort study*—For matched studies, give matching criteria and number of exposed and unexposed *Case-control study*—For matched studies, give matching criteria and the number of controls per case
Variables	7	Clearly define all outcomes, exposures, predictors, potential confounders, and effect modifiers. Give diagnostic criteria, if applicable ✓ Page 7-8
Data sources/ measurement	8*	For each variable of interest, give sources of data and details of methods of assessment (measurement). Describe comparability of assessment methods if there is more than one group ✓ Page 7-8
Bias	9	Describe any efforts to address potential sources of bias ✓ Page 14
Study size	10	Explain how the study size was arrived at ✓ Page 6
Quantitative variables	11	Explain how quantitative variables were handled in the analyses. If applicable, describe which groupings were chosen and why ✓ Page 8
Statistical methods	12	(*a*) Describe all statistical methods, including those used to control for confounding

For peer review only - http://bmjopen.bmj.com/site/about/guidelines.xhtml

		✓ Page 8
		(*b*) Describe any methods used to examine subgroups and interactions<br>✓ Page 8
		(*c*) Explain how missing data were addressed<br>✓ Page 8 + legend of Table 1
		(*d*) *Cohort study*—If applicable, explain how loss to follow-up was addressed<br>*Case-control study*—If applicable, explain how matching of cases and controls was addressed<br>*Cross-sectional study*—If applicable, describe analytical methods taking account of sampling strategy
		(*e*) Describe any sensitivity analyses

Continued on next page

**Results**		
Participants	13*	(a) Report numbers of individuals at each stage of study—eg numbers potentially eligible, examined for eligibility, confirmed eligible, included in the study, completing follow-up, and analysed<br>✓ Page 6
		(b) Give reasons for non-participation at each stage
		(c) Consider use of a flow diagram
Descriptive data	14*	(a) Give characteristics of study participants (eg demographic, clinical, social) and information on exposures and potential confounders<br>✓ Table 1
		(b) Indicate number of participants with missing data for each variable of interest<br>✓ Legend of Table 1
		(c) *Cohort study*—Summarise follow-up time (eg, average and total amount)
Outcome data	15*	*Cohort study*—Report numbers of outcome events or summary measures over time
		Case-control study—Report numbers in each exposure category, or summary measures of exposure
		Cross-sectional study—Report numbers of outcome events or summary measures
Main results	16	(*a*) Give unadjusted estimates and, if applicable, confounder-adjusted estimates and their precision (eg, 95% confidence interval). Make clear which confounders were adjusted for and why they were included<br>Unadjusted estimates are available upon request.
		(*b*) Report category boundaries when continuous variables were categorized<br>✓ Page 9
		(*c*) If relevant, consider translating estimates of relative risk into absolute risk for a meaningful time period
Other analyses	17	Report other analyses done—eg analyses of subgroups and interactions, and sensitivity analyses
**Discussion**		
Key results	18	Summarise key results with reference to study objectives<br>✓ Page 12-13
Limitations	19	Discuss limitations of the study, taking into account sources of potential bias or imprecision. Discuss both direction and magnitude of any potential bias<br>✓ Page 14
Interpretation	20	Give a cautious overall interpretation of results considering objectives, limitations, multiplicity of analyses, results from similar studies, and other relevant evidence

For peer review only

		✓ Page 12-14
Generalisability	21	Discuss the generalisability (external validity) of the study results<br>✓ Page 14
**Other information**		
Funding	22	Give the source of funding and the role of the funders for the present study and, if applicable, for the original study on which the present article is based<br>✓ Page 16

*Give information separately for cases and controls in case-control studies and, if applicable, for exposed and unexposed groups in cohort and cross-sectional studies.

Note: An Explanation and Elaboration article discusses each checklist item and gives methodological background and published examples of transparent reporting. The STROBE checklist is best used in conjunction with this article (freely available on the Web sites of PLoS Medicine at http://www.plosmedicine.org/, Annals of Internal Medicine at http://www.annals.org/, and Epidemiology at http://www.epidem.com/). Information on the STROBE Initiative is available at www.strobe-statement.org.

For peer review only

For peer review only - http://bmjopen.bmj.com/site/about/guidelines.xhtml

BMJ Open

Older adults' willingness to deprescribe and medication changes: A longitudinal sub-study of a cluster randomized controlled trial

Journal:	*BMJ Open*
Manuscript ID	bmjopen-2023-075325.R2
Article Type:	Original research
Date Submitted by the Author:	09-Nov-2023
Complete List of Authors:	Jungo, Katharina Tabea; University of Bern, Institute of Primary Health Care BIHAM<br>Weir, Kristie; University of Bern, Institute of Primary Health Care BIHAM; The University of Sydney, Sydney School of Public Health, Faculty of Medicine and Health<br>Cateau, Damien; University of Lausanne, Community Pharmacy, Centre for Primary Care and Public Health (Unisanté); University of Geneva, School of Pharmaceutical Sciences<br>Streit, Sven; University of Bern, Institute of Primary Health Care BIHAM
**Primary Subject Heading**:	General practice / Family practice
Secondary Subject Heading:	General practice / Family practice, Health services research
Keywords:	Aged, Primary Health Care, Health Services, Chronic Disease, Surveys and Questionnaires

SCHOLARONE™
Manuscripts

BMJ

I, the Submitting Author has the right to grant and does grant on behalf of all authors of the Work (as defined in the below author licence), an exclusive licence and/or a non-exclusive licence for contributions from authors who are: i) UK Crown employees; ii) where BMJ has agreed a CC-BY licence shall apply, and/or iii) in accordance with the terms applicable for US Federal Government officers or employees acting as part of their official duties; on a worldwide, perpetual, irrevocable, royalty-free basis to BMJ Publishing Group Ltd ("BMJ") its licensees and where the relevant Journal is co-owned by BMJ to the co-owners of the Journal, to publish the Work in this journal and any other BMJ products and to exploit all rights, as set out in our licence.

The Submitting Author accepts and understands that any supply made under these terms is made by BMJ to the Submitting Author unless you are acting as an employee on behalf of your employer or a postgraduate student of an affiliated institution which is paying any applicable article publishing charge ("APC") for Open Access articles. Where the Submitting Author wishes to make the Work available on an Open Access basis (and intends to pay the relevant APC), the terms of reuse of such Open Access shall be governed by a Creative Commons licence – details of these licences and which Creative Commons licence will apply to this Work are set out in our licence referred to above.

Other than as permitted in any relevant BMJ Author's Self Archiving Policies, I confirm this Work has not been accepted for publication elsewhere, is not being considered for publication elsewhere and does not duplicate material already published. I confirm all authors consent to publication of this Work and authorise the granting of this licence.

For peer review only

Older adults' willingness to deprescribe and medication changes: A longitudinal sub-study of a cluster randomized controlled trial

Author list: Katharina Tabea Jungo* [1,2], PhD, Kristie Rebecca Weir* [1,3], PhD, Damien Cateau [4,5], PhD, Sven Streit [1], PhD
*shared first

Affiliations
[1] Institute of Primary Health Care BIHAM, University of Bern, Bern, Switzerland
[2] Center for Healthcare Delivery Science and Division of Pharmacoepidemiology and Pharmacoeconomics, Department of Medicine, Brigham and Women's Hospital and Harvard Medical School, Boston, Massachusetts
[3] Sydney School of Public Health, Faculty of Medicine and Health, The University of Sydney, Sydney, Australia
[4] Community Pharmacy, Centre for Primary Care and Public Health (Unisanté), University of Lausanne, Lausanne, Switzerland
[5] School of Pharmaceutical Sciences, University of Geneva, Geneva, Switzerland

Corresponding author:
Katharina Tabea Jungo
Katharina.jungo@protonmail.com
Mittelstrasse 43, 3012 Bern, Institute of Primary Health Care BIHAM, University of Bern, Bern, Switzerland

Word count manuscript: 2,930

Key words (5): polypharmacy, deprescribing, older adults, comorbidity, medications

For peer review only

Abstract

Objective: To investigate the association between older patients' willingness to have one or more medications deprescribed and: (1) change in medications, (2) change in the appropriateness of medications, and (3) implementation of prescribing recommendations generated by the electronic decision support system tested in the 'Optimizing PharmacoTherapy In the Multimorbid Elderly in Primary CAre' (OPTICA) trial.

Design: A longitudinal sub-study of the OPTICA trial, a cluster randomized controlled trial.

Setting: Swiss primary care settings.

Participants: Participants were aged ≥65 years, with ≥3 chronic conditions, and ≥5 regular medications recruited from 43 GP practices.

Exposures: Patients' willingness to have medications deprescribed was assessed using 3 questions from the 'revised Patient Attitudes Towards Deprescribing' (rPATD) questionnaire and its concerns about stopping score.

Measures/Analyses: Medication-related outcomes were collected at 1-year follow-up. Aim 1 outcome: change in the number of long-term medications between baseline and 12-month follow-up. Aim 2 outcome: change in medication appropriateness (Medication Appropriateness Index). Aim 3 outcome: binary variable on whether any prescribing recommendation generated during the OPTICA medication review was implemented. We used multilevel linear regression analyses (Aim 1, Aim 2) and multilevel logistic regression analyses (Aim 3). Models were adjusted for sociodemographic variables and the clustering effect at GP level.

Results: 298 patients completed the rPATD, 45% were women and 78 years was the median age. A statistically significant association was found between the concerns about stopping score and the change in the number of medications over time (per 1-unit increase in the score the average number of medications use was 0.65 higher; 95% CI: 0.08 to 1.22). There was no statistically significant association between patients' willingness to have medications deprescribed and medication-related outcomes.

Conclusions: These findings indicate that there is no association between patients' willingness to have medications deprescribed and medication-related outcomes over one year.

Trial registration: NCT03724539Clinicaltrials.gov NCT03724539

For peer review only - http://bmjopen.bmj.com/site/about/guidelines.xhtml

Strengths and limitations:

- A longitudinal sub-study (n=298 patients) of the OPTICA trial – a cluster randomized controlled trial conducted in Swiss primary care settings.
- Few studies have explored the association between medication-related outcomes after 1 year and patients' hypothetical willingness to have their medications deprescribed as measured by a self-report questionnaire.
- The medication-related outcomes included not only the number of medications between baseline and 12-month follow-up, but also and medication appropriateness (as measured by the Medication Appropriateness Index).
- The longitudinal study design allowed for a clear temporal distinction between patients' willingness to have medications deprescribed assessed at baseline and the medication-related outcomes after 1 year.
- Older adults agreeing to participate in the OPTICA trial could have had a higher willingness to have one or more of their medications deprescribed.

For peer review only - http://bmjopen.bmj.com/site/about/guidelines.xhtml

For peer review only

Introduction

Globally, there is increasing focus on polypharmacy in the older population. Up to 50% of older adults aged 65 years and above take one or more inappropriate medication,(1) which has been associated with harmful effects on health outcomes and quality of life(2, 3). In older patients with multiple chronic conditions (multimorbidity) the percentage is even higher(4). A medication is considered *inappropriate* when potential harms outweigh potential benefits in the individual (5). One strategy to mitigate against inappropriate medication use is deprescribing, the process of reducing or stopping medications that lack benefit or may cause harm(6). However, implementing deprescribing decisions in clinical practice is challenging.

The extensive research into the barriers and facilitators of deprescribing has shown mixed results. Older adults often hold ambivalent attitudes in that they may express a willingness to reduce their medications whilst perceiving all their medications as beneficial and necessary(7, 8). Clinicians can perceive their patients are reluctant to have their medications deprescribed(9, 10). A recent study from Switzerland found a quarter of patients (22/87) declined their GP's offer to deprescribe a medication in a cluster-randomized study – even with a shared decision-making intervention (11). Similarly, a substantial proportion of participants (42% - 75%) decline to participate in deprescribing intervention studies(12-14).

To understand patients' attitudes towards deprescribing, researchers have turned towards self-reported assessments such as the Patient Perceptions of Deprescribing survey(15) and the revised Patients' Attitudes Towards Deprescribing (rPATD) questionnaire(16). The rPATD has high uptake in the deprescribing literature with the global question most frequently used: *"If my doctor said it was possible, I would be willing to stop one or more of my medicines"*. A systematic review of this questionnaire (and related versions) found inconsistency in whether there was statistical significance (and direction of the association) between characteristics and willingness to deprescribe(17). However, mostly cross-sectional surveys were included, and few studies have used the rPATD in longitudinal research or investigating medication-related outcomes such as appropriateness or implementation of deprescribing.

For peer review only - http://bmjopen.bmj.com/site/about/guidelines.xhtml

It remains to be seen if patients' willingness to have medications deprescribed is associated with the implementation of actual deprescribing decisions and real changes in medication-related outcomes over time.

To address this gap in the deprescribing literature we aimed to investigate the association between older adults' willingness to have medications deprescribed and (1) actual change in their medications at 1-year follow-up, (2) change in the appropriateness of medications at 1-year follow-up, and (3) actual implementation of prescribing recommendations generated by an electronic decision support system tested in a clinical trial (OPTICA) to stop medications.

For peer review only

Methods

Overview of the OPTICA trial

The methods and results of the "Optimising PharmacoTherapy In the multimorbid elderly in primary Care" (OPTICA) trial (*clinicaltrials.gov identifier: NCT03724539*) have been reported elsewhere.(18-20) In brief, 323 patients from 43 GP practices were recruited into this cluster randomized clinical trial between January 2019 and February 2020. The 12-month follow-up ended in February 2021. 21 GPs with 160 patients were assigned to the intervention group and 22 GPs with 163 patients to the control group. Eligible patients were 65 years or older, had ≥3 chronic conditions, and were taking ≥5 medications regularly. Baseline willingness to have medications deprescribed was assessed at baseline. While GPs in the control group continued to provide usual care to their patients including a discussion of patients' medications in accordance with their usual practice, GPs in the intervention group performed a structured medication review centred around an electronic clinical decision support system called the "Systematic Tool to Reduce Inappropriate Prescribing"-Assistant (STRIP-Assistant). This tool is based on the STOPP/START criteria and generated prescribing recommendations to stop, start, or adapt the dosage and flagged interactions(21-23). The OPTICA trial had a pragmatic design with data collected from participants' electronic health records (e.g., medications, diagnoses) and from participants or their legal representatives over the phone (e.g., quality of life, living situation, etc.). The two primary outcomes of the trial were the improvement in the Medication Appropriateness Index (MAI) and the Assessment of Underutilization (AOU) at 12 months(24-26). Secondary outcomes included the number of medications, number of falls and

For peer review only - http://bmjopen.bmj.com/site/about/guidelines.xhtml

fractures and quality of life. In the intention-to-treat analysis at 12 months, there were no group differences in the improvement of medication appropriateness (Odds ratio (OR)=1.05; 95% confidence interval (CI)=0.59 to 1.87) nor the number of prescribing omissions (OR=0.90; 95% CI=0.41 to 1.96). The per-protocol analysis showed no statistically significant group difference either and there were no group differences in the secondary outcomes. In 59% of participants at least one prescribing recommendation to stop or start a medication was implemented. It is of note that not all prescribing recommendations generated by STRIPA were accepted by GPs and discussed with patients. The OPTICA trial was approved by the Cantonal Ethics Committee of the Canton of Bern (*BASEC-ID: 2018–00914*). All participants or their legal representatives provided written informed consent.

Study design and sample definition

This is a longitudinal, post-hoc sub-study of data collected during the OPTICA trial. Data from the trial baseline, the 6-month follow-up, and the 12-month follow-up were used for the present analyses. This manuscript adheres to the Strengthening the Reporting of Observational Studies in Epidemiology (STROBE) reporting guideline for observational studies(27). All 323 participants of the OPTICA trial were older adults (≥65 years of age), with multimorbidity (≥3 chronic conditions) and polypharmacy (≥5 medications). We limited the present analyses to the participants for whom the patient version of the 'revised Patient Attitudes Towards Deprescribing' (rPATD) was used (N = 298)(16).

Assessment of patients' willingness to have medications deprescribed

Patients' attitudes towards having medications deprescribed hypothetically was measured using the rPATD at baseline. The rPATD contains 22 questions with "Strongly disagree (1)" and "Strongly agree (5)" as the scale anchors(16). For the main analyses, we used the global question from the rPATD "If my doctor said it was possible, I would be willing to stop one or more of my regular medicines" as the independent variable, which measures patients' willingness to accept deprescribing proposed by a medical doctor. In addition, we used two questions from the rPATD "appropriateness" factor ("I would like to try stopping one of my medicines to see how I feel without

For peer review only - http://bmjopen.bmj.com/site/about/guidelines.xhtml

it" and "I would like my doctor to reduce the dose of one or more of my medicines"), which aim to measure patients' desires to try to stop or reduce medicines (Box 1). Further, we used the rPATD "concerns about stopping" factor score (ranging from 1 to 5) calculated based on five rPATD questions as independent variables. Several questions from the rPATD were used as independent variables given the ceiling effect of the global rPATD willingness to deprescribe question and the fact that there is more variation in the responses to the other two rPATD questions and the concerns about stopping score.

Box 1: Attitudes towards deprescribing: rPATD questions

**Global question:**<br>*"If my doctor said it was possible, I would be willing to stop one or more of my regular medicines"*
**Appropriateness questions:**<br>*"I would like to try stopping one of my medicines to see how I feel without it"*<br>*"I would like my doctor to reduce the dose of one or more of my medicines"*
**Concerns about stopping questions:**<br>*"I have had a bad experience when stopping a medicine before"*<br>*"I would be reluctant to stop a medicine that I had been taking for a long time"*<br>*"If one of my medicines was stopped I would be worried about missing out on future benefits"*<br>*"I get stressed whenever changes are made to my medicines"*<br>*"If my doctor recommended stopping a medicine I would feel that he/she was giving up on me"*

Assessment of medication-related outcomes over time

Medication-related outcomes over time were assessed using data collected at baseline and throughout the follow-up period of the OPTICA trial. Details on how the three medication-related outcomes were assessed – change in the number of medications, medication appropriateness, and the implementation of prescribing recommendations – can be found in **Box 2**.

For peer review only - http://bmjopen.bmj.com/site/about/guidelines.xhtml

Box 2: Assessment of medication-related outcomes

	Outcome	Description	Measurement
**Aim 1**	Number of long-term medications	Integer number of medications used for ≥90 days	Change in the number of long-term medications (≥90 days, "as needed" medications were excluded) between baseline and the 12-month follow-up.
**Aim 2**	Medication appropriateness	The Medication Appropriateness Index (MAI)(28) is an implicit tool for assessing the appropriateness of medication prescribing. The 10-item version of the MAI was used as one of the co-primary outcomes of the OPTICA trial(20), however, the cost effectiveness item was excluded for feasibility reasons. Using data on medications, diagnoses, and lab values the blinded assessors rated the nine remaining criteria of the MAI for each medication used for ≥90 days using a three-point scale ranging from A=appropriate, B=marginally appropriate, to C=inappropriate.	Change in the average medication appropriateness between baseline and the 12-month follow-up. We first calculated the average MAI for the baseline and the 12-month follow-up by dividing the total MAI score of the respective timepoint by the number of long-term medications at this timepoint. Then we calculated the change in the average MAI between baseline and the 12-month follow-up.
**Aim 3**	Implementation of prescribing recommendations to stop medications	Recommendation implemented yes vs. no	Binary variable describing whether any deprescribing recommendation to stop a medication generated by the electronic decision support system tested in the OPTICA trial had been implemented or not at the patient level. Only data from the OPTICA intervention group was used.

For peer review only - http://bmjopen.bmj.com/site/about/guidelines.xhtml

Co-variates

The following variables were used to adjust the analyses: gender, age, educational status, number of chronic conditions, living situation, capable of leaving the house (yes/no), patients' satisfaction with medications, and number of GP visits in the 6 months prior to the study enrolment. The included variables were based on the literature of the factors associated with number of medications/polypharmacy and the factors associated with potentially inappropriate medication use/medication appropriateness considering the data available from the OPTICA trial.(29-35)

Statistical analysis

First, we described the demographics and main clinical characteristics of the study participants. Second, we descriptively analysed three questions from the rPATD and the concerns about stopping score to describe patients' willingness to have medications deprescribed at baseline. Third, we performed a set of multilevel regression analyses. For Aims 1 and 2, we used multilevel linear regression models to investigate the association between patients' willingness to have medications deprescribed and the outcomes. In subgroup analyses, we restricted the analyses to the OPTICA intervention group. For Aim 3, we used a multilevel logistic regression model to investigate the association between patients' willingness to have medications deprescribed and the binary outcome variable. For Aim 3, we used data from the OPTICA intervention group only. All analyses were adjusted for the clustering effect at the GP level and the measurable co-variates listed in the section above plus the group allocation during the trial (except for the analyses for Aim 3, which were based on data from the intervention group only). Analyses were limited to the observed data, and we did not use any multiple imputation methods. All analyses were performed with STATA 15.1 (StataCorp, College Station, TX, USA). A p-value < 0.05 was considered significant.

Patient and public involvement

No patients were involved in this sub-study of the OPTICA trial.

Results

Baseline characteristics

For peer review only - http://bmjopen.bmj.com/site/about/guidelines.xhtml

Table 1 describes the baseline characteristics of study participants**.** Out of the 298 participants for whom information on their willingness to have medications deprescribed was assessed at baseline (92% of all trial participants), 45% were women and the median age was 78 years. 75% (224/298) of the participants had equal or higher than median willingness to have medications deprescribed.

For peer review only

For peer review only - http://bmjopen.bmj.com/site/about/guidelines.xhtml

Table 1. Baseline characteristics of study participants by willingness to deprescribe (n = 298)

	All patients in the sample *(n=298)*[2]	Patients with lower than median willingness to deprescribe *(n=74)*[1]	Patients with equal or higher than median willingness to deprescribe *(n=224)*[1]
Age (in years)	78 (74-83)	79 (74-83)	78 (74-83)
Female	133 (45)	39 (53)	94 (42)
Patient education			
Mandatory schooling or less	113 (38)	25 (34)	86 (39)
Diploma at secondary school level	139 (47)	33 (45)	106 (47)
Higher education diploma	45 (15)	16 (22)	29 (13)
Number of chronic conditions	7 (5-10)	7 (5-9)	7 (5-11)
Living situation			
In apartment/ house without any external help	227 (76)	62 (84)	165 (74)
In apartment/ house with some external help	61 (20)	9 (12)	52 (23)
In a nursing home	10 (3)	3 (4)	7 (3)
Patient is unable to leave the house (as compared to not housebound)	7 (2)	2 (3)	5 (2)
Equal or higher than median satisfaction with current medication use (as compared to lower than medication willingness to deprescribe)	215 (72)	59 (80)	156 (70)
Number of GP consultations during the 6-month follow-up period prior to the enrolment into the study trial	8 (5-14)	9 (6-13)	8 (5-15)
Average Medication Appropriateness Index at baseline	1.7 (0.2-5)	1.8 (0.2-6)	1.7 (0.2-4.7)
Number of long-term medications	8 (5-11)	8 (5-10)	8 (5-11)

For continuous variables the median and the interquartile range (IQR) are presented. For categorial variables frequencies and percentages are presented. | [1] Patients' willingness to have medications deprescribed was measured using the rPATD global question "If my doctor said it was possible, I would be willing to stop one or more of my regular medicines". The median willingness to have medications deprescribed corresponded to "strongly agree" with the rPATD global question. | [2] Among the 298 patients, 146 patients were then randomized to the control group and 152 patients to the intervention group. Among patients with lower than median willingness to deprescribe 36 were randomized to the control group and 38 to the intervention group. Among patients with equal or higher than median willingness to deprescribe 110 were assigned to the control group and 114 were randomized to the intervention group. | Missingness: Gender, and age had 0% missing values. Patient education, living situation, housebound yes/no, patient satisfaction with medications, and the number of chronic medications had less than 3% missing information. The number of chronic conditions and the average Medication Appropriateness Index at baseline had less than 7% missing.

For peer review only - http://bmjopen.bmj.com/site/about/guidelines.xhtml

Proxy measures for patients' willingness to have medications deprescribed

Table 2 shows the descriptive results of the different measures used to assess patients' willingness to have medications deprescribed. More than 85% of participants strongly agreed or agreed with the rPATD global question and only 9% of participants disagreed with this statement, whereas there was slightly more variation in responses to the other two rPATD questions. Approximately 60% of participants reported they would like to try stopping one of their medications to see how they would feel without it, whereas 32% disagreed or strongly disagreed with this statement.

Table 2. Patients' attitudes towards having medications deprescribed at baseline[1] (n=298)

rPATD global question: "If my doctor said it was possible, I would be willing to stop one or more of my regular medicines" frequency (percent)				
Strongly agree	Agree	Unsure	Disagree	Strongly disagree
224 (75)	38 (13)	9 (3)	14 (5)	13 (4)
Alternative measurements of patients' willingness to have medications deprescribed based on the rPATD				
Concerns about stopping score		Mean (SD)	Median (IQR)	
		1.8 (0.8)	1.6 (1-2.4)	
"I would like to try stopping one of my medicines to see how I feel without it"				
Strongly agree	Agree	Unsure	Disagree	Strongly disagree
120 (40)	65 (22)	19 (6)	59 (20)	35 (12)
"I would like my doctor to reduce the dose of one or more of my medicines"				
Strongly agree	Agree	Unsure	Disagree	Strongly disagree
153 (51)	64 (22)	24 (8)	29 (10)	28 (9)

[1] As measured by the 'revised Patients' Attitudes Towards Deprescribing (rPATD) questionnaire. Source: Reeve, E., al. Development and Validation of the Revised Patients' Attitudes Towards Deprescribing (rPATD) Questionnaire: Versions for Older Adults and Caregivers. Drugs Aging 33, 913–928 (2016). | Missingness: There was 0% missingness in rPATD questions and the concerns of stopping score.

Number of medications over time

Table 3 shows the associations between the different measures assessing patients' willingness to have medications deprescribed and the change in number of medications throughout the 12-month follow-up period. At the 12-month follow-up, the mean change in the number of medications was -0.2 (standard deviation=4.2). The only statistically significant association was between the concerns about stopping score and the change in the number of medications (coefficient: 0.65, 95% CI: 0.08 to 1.22). A higher score indicates being more concerned about stopping. Meaning, per 1-unit increase in the concerns about stopping score the change in the number of medications between baseline and the 12-month follow-up increased by 0.65.

For peer review only - http://bmjopen.bmj.com/site/about/guidelines.xhtml

Table 3. Multivariate associations between the change in the number of medications throughout the 12-month follow-up period and patients' willingness to deprescribe (n=253)

Name of the variable	*Coefficient*	*p-value*	*95% confidence interval*
rPATD global question: "If my doctor said it was possible, I would be willing to stop one or more of my regular medicines" (reference: strongly agree)			
agree	-0.96	0.169	-2.33 to 0.41
unsure	0.61	0.963	-2.52 to 2.64
disagree	0.58	0.598	-1.56 to 2.71
strongly disagree	0.26	0.806	-1.81 to 2.33
Alternative measurements of patients' willingness to have medications deprescribed based on the rPATD			
Concerns about stopping score (per 1-unit increase)[1]	0.65	**0.026**	0.08 to 1.22
'I would like to try stopping one of my medicines to see how I feel without it' (reference: strongly agree)			
agree	-0.12	0.830	-1.33 to 1.07
unsure	0.62	0.509	-1.24 to 2.51
disagree	0.47	0.448	-0.74 to 1.68
strongly disagree	-0.21	0.774	-1.68 to 1.25
'I would like my doctor to reduce the dose of one or more of my medicines' (reference: strongly agree)			
agree	1.13	0.070	-0.09 to 2.36
unsure	-0.97	0.251	-2.64 to 0.69
disagree	0.79	0.306	-0.72 to 2.31
strongly disagree	0.71	0.359	-0.81 to 2.24

Multilevel linear regression models adjusted for patient age, education status, gender, number of chronic conditions, living situation, whether the patient is housebound or not, patient satisfaction with medications, the number of GP consultations in the 6-months prior to the study inclusion, and the group allocation during the trial. | [1]As calculated based on Reeve, E., al. Development and Validation of the Revised Patients' Attitudes Towards Deprescribing (rPATD) Questionnaire: Versions for Older Adults and Caregivers. Drugs Aging 33, 913–928 (2016). | Missingness: The change in the number of chronic medications over the 12-month follow-up period had 8% missing values.

Medication appropriateness over time

The associations between patients' willingness to have medications deprescribed and the change in medication appropriateness throughout the 12-month follow-up period is shown in **Table 4**. At the 12-month follow-up, the mean change in the average Medication Appropriateness Index was -0.75 (Standard deviation=2.5). We did not find evidence for the statistically significant associations. In the subgroup analyses restricted to the OPTICA intervention group, we found statistically significant associations between patients' being undecided or (strongly) agreeing with the statement "I would like my doctor to reduce the dose of one or more of my medicines" and an improvement in medication appropriateness between baseline and the 12-month follow-up period (results not presented).

For peer review only - http://bmjopen.bmj.com/site/about/guidelines.xhtml

Table 4. Multivariate associations between the change in the medication appropriateness[1] throughout the 12-month follow-up period and patients' willingness to deprescribe[2] (n=242)

Name of the variable	*Coefficient*	*p-value*	*95% confidence interval*
rPATD global question: "If my doctor said it was possible, I would be willing to stop one or more of my regular medicines" (reference: strongly agree)			
agree	0.35	0.426	-0.51 to 1.21
unsure	0.92	0.293	-0.79 to 2.63
disagree	-1.01	0.145	-2.36 to 0.35
strongly disagree	-0.80	0.221	-2.08 to 0.48
Alternative measurements of patients' willingness to have medications deprescribed based on the rPATD			
Concerns about stopping score (per 1-unit increase)[1]	-0.29	0.120	-0.65 to 0.08
'I would like to try stopping one of my medicines to see how I feel without it' (reference: strongly agree)			
agree	-0.45	0.253	-1.21 to 0.32
unsure	-0.66	0.281	-1.87 to 0.54
disagree	-0.45	0.246	-1.22 to 0.31
strongly disagree	-0.57	0.233	-1.51 to 0.37
'I would like my doctor to reduce the dose of one or more of my medicines' (reference: strongly agree)			
agree	-0.44	0.253	-1.20 to 0.32
unsure	-0.59	0.282	-1.67 to 0.49
disagree	-0.02	0.968	-0.95 to 0.99
strongly disagree	0.13	0.795	-0.85 to 1.11

Multilevel linear regression models adjusted for patient age, education status, gender, number of chronic conditions, living situation, whether the patient is housebound or not, patient satisfaction with medications, the number of GP consultations in the 6-months prior to the study inclusion, and the group allocation during the trial. | [1]As assessed using the Medication Appropriateness Index: Samsa GP, Hanlon JT, Schmader KE, Weinberger M, Clipp EC, Uttech KM, Lewis IK, Landsman PB, Cohen HJ. A summated score for the medication appropriateness index: development and assessment of clinimetric properties including content validity. J Clin Epidemiol. 1994 Aug;47(8):891-6. | [2]As calculated based on Reeve, E., al. Development and Validation of the Revised Patients' Attitudes Towards Deprescribing (rPATD) Questionnaire: Versions for Older Adults and Caregivers. Drugs Aging 33, 913–928 (2016). | Missingness: The change in the Medication Appropriateness Index over the 12-month follow-up period had 13% values.

Implementation of prescribing recommendations

Table 5 shows the association between patients' willingness to have medications deprescribed and the implementation of prescribing recommendations that were generated as part of the OPTICA medication review intervention (n=31). On average, 1 prescribing recommendation to stop or start a medication were reported to be implemented per patient (reported elsewhere (36)) and 59% of patients in the intervention group had 1 or more prescribing recommendation implemented. We did not find any evidence for a statistically significant association between patients' willingness to have medications deprescribed and the implementation of deprescribing recommendations.

For peer review only - http://bmjopen.bmj.com/site/about/guidelines.xhtml

Table 5. Multivariate associations between the implementation of recommendations to stop medications and patients' willingness to deprescribe[1] (n=31)

Name of the variable	Odds ratio	*p-value*	*95% confidence interval*
rPATD global question: "If my doctor said it was possible, I would be willing to stop one or more of my regular medicines" (reference: equal or higher than median agreement)[2]			
Below median agreement	4.90	0.244	0.34 to 71.3
Alternative measurements of patients' willingness to have medications deprescribed based on the rPATD			
Concerns about stopping score (per 1-unit increase)	1.13	0.812	0.41 to 3.13
'I would like to try stopping one of my medicines to see how I feel without it' (reference: equal or higher than median agreement)[2]			
Below median agreement	2.53	0.305	0.43 to 14.89
'I would like my doctor to reduce the dose of one or more of my medicines' (reference: equal or higher than median agreement) [2]			
Below median agreement	7.82	0.086	0.75 to 82.2

[1] Multilevel logistic regression models adjusted for patient age, and gender. | [2] Due to the low number of observations for which the implementation of recommendations was reported, the rPATD question was dichotomized.
The analyses presented in this table used data from the OPTICA intervention group only. Despite several reminders, only a couple of general practitioners from the OPTCIA intervention group reported this information.

For peer review only

Discussion

In this sub-study of a cluster randomised clinical trial, we examined the association between older adults' hypothetical willingness to have one or more medications deprescribed and change in a participant's medications, appropriateness of their medications, and actual implementation of prescribing recommendations. Overall, we found that these medication-related outcomes measured over one year were not associated with the rPATD deprescribing questions measured in this study. To consider reasons why no association was found, firstly we discuss the rPATD questions in more detail and their ability to measure self-reported attitudes towards deprescribing. Secondly, consideration is given to our deprescribing intervention and how medication-related outcomes were measured in this study.

In our study, 88% of participants agreed or strongly agreed with the rPATD global question: "If my doctor said it was possible, I would be willing to have one or more of my medications deprescribed". However, this high agreement was not associated with changes in medication-related outcomes over time. Other deprescribing intervention trials using the rPATD global question(37-39) also found high agreement with hypothetical willingness to deprescribe (86-95%) with no effect on deprescribing or medication-related outcomes. The majority of studies using the rPATD global deprescribing question report greater than 80% agreement(8) therefore, it may be difficult to find an association given the ceiling effect of this question.

For peer review only - http://bmjopen.bmj.com/site/about/guidelines.xhtml

A recent cluster randomised controlled trial conducted in Ireland with older adults taking ≥15 medications found that a higher willingness to deprescribe measured by the rPATD was not only associated with a higher rate of deprescribing but also initiating medicines(17). The authors note that the rPATD global question may identify participants who are agreeable to any medication-changes if they are suggested by their doctor. Supporting this, there is variation between the global question and another rPATD deprescribing question which does not refer to the doctor(8). In our study, agreement was much higher for the rPATD global deprescribing question with 88% of participants willing to deprescribe if their doctor said it was possible, however 62% wanted to try stopping one of their medications to see how they would feel without it. Other studies using the rPATD have shown substantial differences (30-73% gap) between these questions with the global question responses always higher(8, 40-44) suggesting the influence of the doctor should not be underestimated. Similarly, a content analysis including over 2,500 participants from Australia, the UK and the US found that approximately one-half of older adults who agreed with deprescribing in a hypothetical scenario felt that the doctor's recommendation was an important consideration(45).

There is a complex interplay of factors, such as clinical decision-making and patients' attitudes, that are behind acceptance (or not) of deprescribing. It is possible that the lack of association between the rPATD and medication-related changes in our study was due to the inconclusive effectiveness of the OPTICA deprescribing intervention, which is similar to other deprescribing interventional studies. While it is useful to quantify attitudes towards deprescribing to get a sense of older adults' general thoughts about their medications, it may be unfair to expect self-reported attitudes to equate to actual medication changes. Of note, an exploratory deprescribing controlled trial conducted in Australia measured the original PATD (10 questions) at baseline and again at follow-up(46). Although the PATD baseline scores did not predict deprescribing outcomes, statistically significant changes were shown in 3 questions which signalled a shift in patients' beliefs about medicines.

Deprescribing in clinical practice and interventional studies may not occur for many reasons, such as if the general practitioner chooses not to initiate it. From the main OPTICA trial, the most common reasons for not implementing prescribing recommendations were that general practitioners thought that patients' current medications were beneficial and that the recommended change was not suitable. The first study to focus on older adults from multiple countries who disagree with a

For peer review only - http://bmjopen.bmj.com/site/about/guidelines.xhtml

deprescribing recommendation in a vignette-based survey (n=899)(47) found that older adults reported valuing their medications, they expressed doubts about deprescribing, and preferred to avoid change. Respondents who disagreed with the deprescribing recommendation, as opposed to those who strongly disagreed, were more interested in alternative strategies such as improved communication or a replacement medication. Further to this, respondents reported different factors for disagreeing with a deprescribing recommendation based on the medication type (lansoprazole vs simvastatin). Taken together, attitudinal measures of deprescribing may benefit from greater sensitivity to reluctance towards deprescribing, less vulnerability to the doctor's influence, and capturing attitudes towards specific medications(44, 48). Ultimately, it would be useful for a tool to identify patients at different degrees of willingness to deprescribe so that deprescribing interventions can be tailored to their needs and preferences. Further exploration is needed into the link between attitudes towards medicines and actual medication changes, possibly through process evaluations of deprescribing trials.

Strengths and Limitations

The present analyses were strengthened by the longitudinal design, which allows for a clear temporal distinction between patients' willingness to have medications deprescribed assessed at baseline and the medication-related outcomes over time. Additionally, the intervention to optimize medication was offered randomly. We would like to emphasize the following limitations of these present analyses. Firstly, patients agreeing to participate in the OPTICA trial could have had a higher willingness to have one or more of their medications deprescribed. Some patients were excluded from the analyses due to missing data on their medication. Also, to determine the medication-related outcomes for aim 1 and 2 we used prescribing data from electronic health records, which does not necessarily correspond to what medications were actually used by patients. Finally, despite several reminders, only a small proportion of GPs from the intervention group reported which prescribing recommendations were implemented together with patients. This explains the smaller sample size for our third aim. Due to the small sample size used to analyse Aim 3, the confidence intervals were wide and imprecise.

For peer review only - http://bmjopen.bmj.com/site/about/guidelines.xhtml

Conclusions

Our findings indicate that there is no association between patients' willingness to have medications deprescribed and medication-related outcomes over time. It is important to capture a range of participant attitudes that are both for and against deprescribing, as well as to consider the relationship between self-report surveys and actual deprescribing. The results highlight the need for further research to better understand the factors that contribute to successful deprescribing in primary care settings.

For peer review only

For peer review only - http://bmjopen.bmj.com/site/about/guidelines.xhtml

Declarations

Data sharing statement

We will make the data for this study available to other researchers upon request after publication. The data will be made available for scientific research purposes, after the proposed analysis plan has been approved. Data and documentation will be made available through a secure file exchange platform after approval of the proposal. In addition, a data transfer agreement must be signed (which defines obligations that the data requester must adhere to with regard to privacy and data handling). Deidentified participant data limited to the data used for the proposed project will be made available, along with a data dictionary and annotated case report forms. For data access, please contact the corresponding author.

Ethical approval

The study protocol of the OPTICA trial and other documentation was approved by the competent ethics committee of the canton of Bern (KEK), Switzerland, and the Swiss regulatory authority (Swissmedic) (BASEC ID: 2018–00914). The KEK and Swissmedic received annually safety reports and were informed about the end of the study. All participants gave their written informed consent. The OPTICA trial was performed in accordance with relevant regulations and guidelines.

Funding

The OPTICA trial was supported by the Swiss National Science Foundation, within the framework of the National Research Programme 74 "Smarter Health Care" (NRP74) under contract number 407440_167465 (to Prof Streit, Prof Rodondi, and Prof Schwenkglenks). Dr Jungo was funded by a Postdoc.Mobility Fellowship from the Swiss National Science Foundation (P500PM_206728). Dr Weir was funded by a Swiss Government Excellence Scholarship, a Swiss National Science Foundation Scientific Exchanges grant and a NHMRC Emerging Leader Research Fellowship (2017295).

For peer review only - http://bmjopen.bmj.com/site/about/guidelines.xhtml

Role of the Funder

The funders had no role in the design and conduct of the study; collection, management, analysis, and interpretation of the data; preparation, review, or approval of the manuscript; and decision to submit the manuscript for publication.

Conflict of interest disclosures

The authors do not have any conflicts of interest to disclose.

Acknowledgements

We would like to thank the GPs who participated in the OPTICA trial for their great contributions without which it would have been impossible to conduct this study. We also thank the participants for consenting to participate in our study. Dr Jungo was a member of the Junior Investigator Intensive Program of the US Deprescribing Research Network, which is funded by the National Institute on Aging.

Author contributions

All authors contributed to the concept and study design. All authors contributed to the acquisition, analysis, or interpretation of data. KTJ and KRW wrote the first draft of the manuscript. All other authors provided feedback and approved the final version of the manuscript. KTJ and SS provided administrative and technical support. KTJ did the statistical analyses. SS obtained funding for the work and supervised the study. KTJ and KRW had full access to all the data in the study and are the guarantors.

For peer review only

References

1. Maher RL, Hanlon J, Hajjar ER. Clinical consequences of polypharmacy in elderly. Expert Opinion on Drug Safety. 2014;13(1):57-65.
2. Fried TR, O'Leary J, Towle V, Goldstein MK, Trentalange M, Martin DK. Health outcomes associated with polypharmacy in community-dwelling older adults: a systematic review. J Am Geriatr Soc. 2014;62(12):2261-72.
3. Kantor ED, Rehm CD, Haas JS, Chan AT, Giovannucci EL. Trends in Prescription Drug Use Among Adults in the United States From 1999-2012. Jama. 2015;314(17):1818-31.
4. Jungo KT, Streit S, Lauffenburger JC. Utilization and Spending on Potentially Inappropriate Medications by US Older Adults with Multiple Chronic Conditions using Multiple Medications. Archives of Gerontology and Geriatrics. 2021;93:104326.
5. American Geriatrics Society 2019 Updated AGS Beers Criteria® for Potentially Inappropriate Medication Use in Older Adults. J Am Geriatr Soc. 2019;67(4):674-94.
6. Scott IA, Hilmer SN, Reeve E, Potter K, Le Couteur D, Rigby D, et al. Reducing Inappropriate Polypharmacy: The Process of DeprescribingReducing Inappropriate PolypharmacyReducing Inappropriate Polypharmacy. JAMA Internal Medicine. 2015;175(5):827-34.
7. Weir K, Nickel B, Naganathan V, Bonner C, McCaffery K, Carter SM, et al. Decision-Making Preferences and Deprescribing: Perspectives of Older Adults and Companions About Their Medicines. J Gerontol B Psychol Sci Soc Sci. 2018;73(7):e98-e107.
8. Weir KR, Ailabouni NJ, Schneider CR, Hilmer SN, Reeve E. Consumer attitudes towards deprescribing: a systematic review and meta-analysis. The journals of gerontology Series A, Biological sciences and medical sciences. 2021.
9. Weir KR, Naganathan V, Carter SM, Tam CWM, McCaffery K, Bonner C, et al. The role of older patients' goals in GP decision-making about medicines: a qualitative study. BMC Family Practice. 2021;22(1):13.
10. Ailabouni NJ, Nishtala PS, Mangin D, Tordoff JM. General practitioners' insight into deprescribing for the multimorbid older individual: a qualitative study. International Journal of Clinical Practice. 2016;70(3):261-76.
11. Zechmann S, Senn O, Valeri F, Essig S, Merlo C, Rosemann T, et al. Effect of a patient-centred deprescribing procedure in older multimorbid patients in Swiss primary care - A cluster-randomised clinical trial. BMC geriatrics. 2020;20(1):471.
12. Kutner JS, Blatchford PJ, Taylor DH, Ritchie CS, Bull JH, Fairclough DL, et al. Safety and benefit of discontinuing statin therapy in the setting of advanced, life-limiting illness: a randomized clinical trial. JAMA internal medicine. 2015;175(5):691-700.
13. McCarthy C, Flood M, Clyne B, Smith SM, Boland F, Wallace E, et al. Association between patient attitudes towards deprescribing and subsequent prescription changes. Basic & Clinical Pharmacology & Toxicology. 2023.
14. Vasilevskis EE, Shah AS, Hollingsworth EK, Shotwell MS, Kripalani S, Mixon AS, et al. Deprescribing Medications Among Older Adults From End of Hospitalization Through Postacute Care: A Shed-MEDS Randomized Clinical Trial. JAMA Internal Medicine. 2023;183(3):223-31.
15. Linsky AM, Stolzmann K, Meterko M. The Patient Perceptions of Deprescribing (PPoD) Survey: Short-Form Development. Drugs Aging. 2020;37(12):909-16.
16. Reeve E, Low LF, Shakib S, Hilmer SN. Development and Validation of the Revised Patients' Attitudes Towards Deprescribing (rPATD) Questionnaire: Versions for Older Adults and Caregivers. Drugs Aging. 2016;33(12):913-28.
17. McCarthy C, Flood M, Clyne B, Smith SM, Boland F, Wallace E, et al. Association between patient attitudes towards deprescribing and subsequent prescription changes (preprint). 2023.
18. Jungo KT, Rozsnyai Z, Mantelli S, Floriani C, Löwe AL, Lindemann F, et al. 'Optimising PharmacoTherapy In the multimorbid elderly in primary CAre' (OPTICA) to improve medication appropriateness: study protocol of a cluster randomised controlled trial. BMJ open. 2019;9(9):e031080.
19. Jungo KT, Meier R, Valeri F, Schwab N, Schneider C, Reeve E, et al. Baseline characteristics and comparability of older multimorbid patients with polypharmacy and general practitioners participating in a randomized controlled primary care trial. BMC Family Practice. 2021;22(1):123.

For peer review only - http://bmjopen.bmj.com/site/about/guidelines.xhtml

20. Jungo KT, Ansorg A-K, Floriani C, Rozsnyai Z, Schwab N, Meier R, et al. Optimising prescribing in older adults with multimorbidity and polypharmacy in primary care (OPTICA): cluster randomised clinical trial. bmj. 2023;381.
21. Drenth-van Maanen AC, Leendertse AJ, Jansen PAF, Knol W, Keijsers C, Meulendijk MC, et al. The Systematic Tool to Reduce Inappropriate Prescribing (STRIP): Combining implicit and explicit prescribing tools to improve appropriate prescribing. J Eval Clin Pract. 2018;24(2):317-22.
22. Meulendijk MC, Spruit MR, Drenth-van Maanen AC, Numans ME, Brinkkemper S, Jansen PAF, et al. Computerized Decision Support Improves Medication Review Effectiveness: An Experiment Evaluating the STRIP Assistant's Usability. Drugs & Aging. 2015;32(6):495-503.
23. Meulendijk MC, Spruit MR, Willeboordse F, Numans ME, Brinkkemper S, Knol W, et al. Efficiency of Clinical Decision Support Systems Improves with Experience. Journal of medical systems. 2016;40(4):76.
24. Hanlon JT, Schmader KE, Ruby CM, Weinberger M. Suboptimal prescribing in older inpatients and outpatients. J Am Geriatr Soc. 2001;49(2):200-9.
25. Hanlon JT, Schmader KE, Samsa GP, Weinberger M, Uttech KM, Lewis IK, et al. A method for assessing drug therapy appropriateness. Journal of clinical epidemiology. 1992;45(10):1045-51.
26. Samsa GP, Hanlon JT, Schmader KE, Weinberger M, Clipp EC, Uttech KM, et al. A summated score for the medication appropriateness index: development and assessment of clinimetric properties including content validity. Journal of clinical epidemiology. 1994;47(8):891-6.
27. von Elm E, Altman DG, Egger M, Pocock SJ, Gøtzsche PC, Vandenbroucke JP. The Strengthening the Reporting of Observational Studies in Epidemiology (STROBE) statement: guidelines for reporting observational studies. Journal of clinical epidemiology. 2008;61(4):344-9.
28. Samsa GP, Hanlon JT, Schmader KE, Weinberger M, Clipp EC, Uttech KM, et al. A summated score for the medication appropriateness index: development and assessment of clinimetric properties including content validity. Journal of clinical epidemiology. 1994;47(8):891-6.
29. Cho HJ, Chae J, Yoon SH, Kim DS. Factors related to polypharmacy and hyper-polypharmacy for the elderly: A nationwide cohort study using National Health Insurance data in South Korea. Clinical and Translational Science. 2023;16(2):193-205.
30. Delara M, Murray L, Jafari B, Bahji A, Goodarzi Z, Kirkham J, et al. Prevalence and factors associated with polypharmacy: a systematic review and meta-analysis. BMC geriatrics. 2022;22(1):601.
31. Nothelle SK, Sharma R, Oakes A, Jackson M, Segal JB. Factors associated with potentially inappropriate medication use in community-dwelling older adults in the United States: a systematic review. International Journal of Pharmacy Practice. 2019;27(5):408-23.
32. Slater N, White S, Venables R, Frisher M. Factors associated with polypharmacy in primary care: a cross-sectional analysis of data from The English Longitudinal Study of Ageing (ELSA). BMJ open. 2018;8(3):e020270.
33. Tian F, Chen Z, Zeng Y, Feng Q, Chen X. Prevalence of Use of Potentially Inappropriate Medications Among Older Adults Worldwide: A Systematic Review and Meta-Analysis. JAMA Network Open. 2023;6(8):e2326910-e.
34. Xu Z, Liang X, Zhu Y, Lu Y, Ye Y, Fang L, et al. Factors associated with potentially inappropriate prescriptions and barriers to medicines optimisation among older adults in primary care settings: a systematic review. Family Medicine and Community Health. 2021;9(4).
35. Ye L, Yang-Huang J, Franse CB, Rukavina T, Vasiljev V, Mattace-Raso F, et al. Factors associated with polypharmacy and the high risk of medication-related problems among older community-dwelling adults in European countries: a longitudinal study. BMC geriatrics. 2022;22(1):1-13.
36. Jungo KT, Ansorg A-K, Floriani C, Rozsnyai Z, Schwab N, Meier R, et al. Optimizing Prescribing in Older Adults with Multimorbidity and Polypharmacy in Primary Care: A Cluster Randomized Clinical Trial (OPTICA Trial). medRxiv. 2022:2022.10.31.22281164.
37. Kouladjian O'Donnell L, Gnjidic D, Sawan M, Reeve E, Kelly PJ, Chen TF, et al. Impact of the Goal-directed Medication Review Electronic Decision Support System on Drug Burden Index: A cluster-randomised clinical trial in primary care. Br J Clin Pharmacol. 2020.
38. Anderson K, Freeman C, Foster M, Scott I. GP-Led Deprescribing in Community-Living Older Australians: An Exploratory Controlled Trial. J Am Geriatr Soc. 2020;68(2):403-10.
39. Turner JP, Martin P, Zhang YZ, Tannenbaum C. Patients beliefs and attitudes towards deprescribing: Can deprescribing success be predicted? Research in social & administrative pharmacy : RSAP. 2020;16(4):599-604.

For peer review only - http://bmjopen.bmj.com/site/about/guidelines.xhtml

40. Rozsnyai Z, Jungo KT, Reeve E, Poortvliet RKE, Rodondi N, Gussekloo J, et al. What do older adults with multimorbidity and polypharmacy think about deprescribing? The LESS study - a primary care-based survey. BMC geriatrics. 2020;20(1):435.
41. Navid P, Nguyen L, Jaber D, Zarzuela K, Musse M, Lu Wang M, et al. Attitudes toward deprescribing among adults with heart failure with preserved ejection fraction. J Am Geriatr Soc. 2021;69(7):1948-55.
42. Crutzen S, Abou J, Smits SE, Baas G, Hugtenburg JG, Heringa M, et al. Older people's attitudes towards deprescribing cardiometabolic medication. BMC geriatrics. 2021;21(1):366.
43. Lukacena KM, Keck JW, Freeman PR, Harrington NG, Huffmyer MJ, Moga DC. Patients' attitudes toward deprescribing and their experiences communicating with clinicians and pharmacists. Therapeutic advances in drug safety. 2022;13:20420986221116465.
44. Bužančić I, Dragović P, Pejaković TI, Markulin L, Ortner-Hadžiabdić M. Exploring Patients' Attitudes Toward Deprescribing and Their Perception of Pharmacist Involvement in a European Country: A Cross-Sectional Study. Patient preference and adherence. 2021;15:2197-208.
45. Vordenberg SE, Rana R, Shang J, Choi J, Scherer AM, Weir KR. Reasons why older adults in three countries agreed with a deprescribing recommendation in a hypothetical vignette. Basic & clinical pharmacology & toxicology. 2023.
46. Anderson K, Freeman C, Foster M, Scott I. GP-led deprescribing in community-living older Australians: an exploratory controlled trial. Journal of the American Geriatrics Society. 2020;68(2):403-10.
47. Weir KR, Shang J, Choi J, Rana R, Vordenberg SE. Factors Important to Older Adults Who Disagree With a Deprescribing Recommendation. JAMA network open. 2023;6(10):e2337281-e.
48. Lüthold RV, Jungo KT, Weir KR, Geier A-K, Scholtes B, Kurpas D, et al. Understanding older patients' willingness to have medications deprescribed in primary care: a protocol for a cross-sectional survey study in nine European countries. BMC Geriatrics. 2022;22(1):920.

For peer review only

For peer review only - http://bmjopen.bmj.com/site/about/guidelines.xhtml

STROBE Statement—checklist of items that should be included in reports of observational studies

	Item No	Recommendation
**Title and abstract**	1	(*a*) Indicate the study's design with a commonly used term in the title or the abstract<br>✓ Page 2
		(*b*) Provide in the abstract an informative and balanced summary of what was done and what was found<br>✓ Page 2
**Introduction**		
Background/rationale	2	Explain the scientific background and rationale for the investigation being reported ✓ Page 4
Objectives	3	State specific objectives, including any prespecified hypotheses
**Methods**		
Study design	4	Present key elements of study design early in the paper<br>✓ Page 5
Setting	5	Describe the setting, locations, and relevant dates, including periods of recruitment, exposure, follow-up, and data collection<br>✓ Page 5-6
Participants	6	(*a*) *Cohort study*—Give the eligibility criteria, and the sources and methods of selection of participants. Describe methods of follow-up<br>*Case-control study*—Give the eligibility criteria, and the sources and methods of case ascertainment and control selection. Give the rationale for the choice of cases and controls<br>*Cross-sectional study*—Give the eligibility criteria, and the sources and methods of selection of participants
		(*b*) *Cohort study*—For matched studies, give matching criteria and number of exposed and unexposed<br>*Case-control study*—For matched studies, give matching criteria and the number of controls per case
Variables	7	Clearly define all outcomes, exposures, predictors, potential confounders, and effect modifiers. Give diagnostic criteria, if applicable<br>✓ Page 7-8
Data sources/ measurement	8*	For each variable of interest, give sources of data and details of methods of assessment (measurement). Describe comparability of assessment methods if there is more than one group<br>✓ Page 7-8
Bias	9	Describe any efforts to address potential sources of bias<br>✓ Page 14
Study size	10	Explain how the study size was arrived at<br>✓ Page 6
Quantitative variables	11	Explain how quantitative variables were handled in the analyses. If applicable, describe which groupings were chosen and why<br>✓ Page 8
Statistical methods	12	(*a*) Describe all statistical methods, including those used to control for confounding

For peer review only

		✓ Page 8
		(*b*) Describe any methods used to examine subgroups and interactions<br>✓ Page 8
		(*c*) Explain how missing data were addressed<br>✓ Page 8 + legend of Table 1
		(*d*) *Cohort study*—If applicable, explain how loss to follow-up was addressed<br>*Case-control study*—If applicable, explain how matching of cases and controls was addressed<br>*Cross-sectional study*—If applicable, describe analytical methods taking account of sampling strategy
		(*e*) Describe any sensitivity analyses

Continued on next page

**Results**		
Participants	13*	(a) Report numbers of individuals at each stage of study—eg numbers potentially eligible, examined for eligibility, confirmed eligible, included in the study, completing follow-up, and analysed<br>✓ Page 6
		(b) Give reasons for non-participation at each stage
		(c) Consider use of a flow diagram
Descriptive data	14*	(a) Give characteristics of study participants (eg demographic, clinical, social) and information on exposures and potential confounders<br>✓ Table 1
		(b) Indicate number of participants with missing data for each variable of interest<br>✓ Legend of Table 1
		(c) *Cohort study*—Summarise follow-up time (eg, average and total amount)
Outcome data	15*	*Cohort study*—Report numbers of outcome events or summary measures over time
		Case-control study—Report numbers in each exposure category, or summary measures of exposure
		Cross-sectional study—Report numbers of outcome events or summary measures
Main results	16	(*a*) Give unadjusted estimates and, if applicable, confounder-adjusted estimates and their precision (eg, 95% confidence interval). Make clear which confounders were adjusted for and why they were included<br>Unadjusted estimates are available upon request.
		(*b*) Report category boundaries when continuous variables were categorized<br>✓ Page 9
		(*c*) If relevant, consider translating estimates of relative risk into absolute risk for a meaningful time period
Other analyses	17	Report other analyses done—eg analyses of subgroups and interactions, and sensitivity analyses
**Discussion**		
Key results	18	Summarise key results with reference to study objectives<br>✓ Page 12-13
Limitations	19	Discuss limitations of the study, taking into account sources of potential bias or imprecision. Discuss both direction and magnitude of any potential bias<br>✓ Page 14
Interpretation	20	Give a cautious overall interpretation of results considering objectives, limitations, multiplicity of analyses, results from similar studies, and other relevant evidence

For peer review only - http://bmjopen.bmj.com/site/about/guidelines.xhtml

		✓ Page 12-14
Generalisability	21	Discuss the generalisability (external validity) of the study results<br>✓ Page 14
**Other information**		
Funding	22	Give the source of funding and the role of the funders for the present study and, if applicable, for the original study on which the present article is based<br>✓ Page 16

*Give information separately for cases and controls in case-control studies and, if applicable, for exposed and unexposed groups in cohort and cross-sectional studies.

Note: An Explanation and Elaboration article discusses each checklist item and gives methodological background and published examples of transparent reporting. The STROBE checklist is best used in conjunction with this article (freely available on the Web sites of PLoS Medicine at http://www.plosmedicine.org/, Annals of Internal Medicine at http://www.annals.org/, and Epidemiology at http://www.epidem.com/). Information on the STROBE Initiative is available at www.strobe-statement.org.

For peer review only

For peer review only - http://bmjopen.bmj.com/site/about/guidelines.xhtml
